# Continuous exposure to isoprenaline reduced myotube size by delaying myoblast differentiation and fusion through the NFAT-MEF2C signaling pathway

**DOI:** 10.1038/s41598-022-22330-w

**Published:** 2023-01-09

**Authors:** Jing Yue, Wei Xu, Li Xiang, Shao-juan Chen, Xin-yuan Li, Qian Yang, Ruo-nan Zhang, Xin Bao, Yan Wang, MagdaleenaNaemi Mbadhi, Yun Liu, Lu-yuan Yao, Long Chen, Xiao-ying Zhao, Chang-qing Hu, Jing-xuan Zhang, Hong-tao Zheng, Yan Wu, Shi-You Chen, Shan Li, Jing Lv, Liu-liu Shi, Jun-ming Tang

**Affiliations:** 1 Department of Physiology, Faculty of Basic Medical Sciences, Hubei University of Medicine, Shiyan, 442000 Hubei People’s Republic of China; 2 Continuing Education Department, Affiliated Hospital of Guilin Medical University, Guilin, 541000 Guangxi People’s Republic of China; 3 Hubei Key Laboratory of Embryonic Stem Cell Research and Institute of Biomedicine, Hubei University of Medicine, Shiyan, 442000 Hubei China; 4 Department of Stomatology, Taihe Hospital, Hubei University of Medicine, Shiyan, 442000 Hubei People’s Republic of China; 5 Department of Physiology, Faculty of Basic Medical Sciences, Zunyi Medical University, Zunyi, 563006 Guizhou People’s Republic of China; 6 Experimental Medical Center, Dongfeng Hospital, Hubei University of Medicine, Shiyan, China; 7 Department of Surgery, University of Missouri, Columbia, USA; 8 Department of Biochemistry, Faculty of Basic Medical Sciences, Hubei University of Medicine, Shiyan, 442000 Hubei People’s Republic of China; 9 Department of Anesthesiology, Taihe Hospital, Hubei University of Medicine, Shiyan, 442000 Hubei People’s Republic of China

**Keywords:** Differentiation, Stem cells

## Abstract

We aimed to explore whether superfluous sympathetic activity affects myoblast differentiation, fusion, and myofiber types using a continuous single-dose isoprenaline exposure model in vitro and to further confirm the role of distinct NFATs in ISO-mediated effects. Compared with delivery of single and interval single, continuous single-dose ISO most obviously diminished myotube size while postponing myoblast differentiation/fusion in a time- and dose-dependent pattern, accompanied by an apparent decrease in nuclear NFATc1/c2 levels and a slight increase in nuclear NFATc3/c4 levels. Overexpression of NFATc1 or NFATc2, particularly NFATc1, markedly abolished the inhibitory effects of ISO on myoblast differentiation/fusion, myotube size and Myh7 expression, which was attributed to a remarkable increase in the nuclear NFATc1/c2 levels and a reduction in the nuclear NFATc4 levels and the associated increase in the numbers of MyoG and MEF2C positive nuclei within more than 3 nuclei myotubes, especially in MEF2C. Moreover, knockdown of NFATc3 by shRNA did not alter the inhibitory effect of ISO on myoblast differentiation/fusion or myotube size but partially recovered the expression of Myh7, which was related to the slightly increased nuclear levels of NFATc1/c2, MyoG and MEF2C. Knockdown of NFATc4 by shRNA prominently increased the number of MyHC +, MyoG or MEF2C + myoblast cells with 1 ~ 2 nuclei, causing fewer numbers and smaller myotube sizes. However, NFATc4 knockdown further deteriorated the effects of ISO on myoblast fusion and myotube size, with more than 5 nuclei and Myh1/2/4 expression, which was associated with a decrease in nuclear NFATc2/c3 levels. Therefore, ISO inhibited myoblast differentiation/fusion and myotube size through the NFAT-MyoG-MEF2C signaling pathway.

## Introduction

Muscular dystrophy (MD) is a group of diseases that cause progressive weakness and loss of muscle mass^1^, at least including Duchenne and Becker MD. The pathology of MD results from intrinsic causes, which include abnormal gene function, and possibly extrinsic causes, such as dysfunction of the autonomous system^2^. However, patients of Duchenne and Becker MD have continuously increased sympathetic activity, which often leads to the progression of dilated cardiomyopathy, arrhythmia, and sudden cardiac death. Nevertheless, previous studies have only primarily focused on the influence of an excessive sympathetic nervous system (SNS) on heart and muscular atrophy, but there is little interest in its probable role in the progression of skeletal myopathy^2–6^.

Muscle satellite cells are conducive to physiological self-renewal and the repair of pathological injury^7^. The pathology of excessive SNS activates β1-AdR, desensitizes β2-AdR and diminishes skeletal muscle anabolism, which further aggravates the loss of muscle mass and muscle weakness^8^. A recent study has shown that satellite cells can express β-AdRs, exhibiting disordered activity changes in β1/β2-AdRs when stimulated with continuous single-dose ISO, triggering cessation or decrease of myoblast differentiation and fusion and myotube size^9^. However, the mechanism of these changes is still unclear.

Generally, stimulated adrenergic receptors (AdRs) coupled with the activation of protein kinase A (PKA) are one of the downstream signaling molecules of activated G-protein/AC^10^. Activated PKA has been linked to the unique phenomenon of myoblast differentiation/fusion and myotube formation, ascribing to alterations in PKA regulatory subunit I (PKA RI) and PKA RII under normal differentiation conditions, especially changes in the PKA RI and PKA RII ratio^11^. Our previous study showed that ISO decreased the ratio of PKA RI/RII in myoblast cells, resulting in the postponement of myoblast differentiation and fusion^9^. Further evidence has shown that PKA phosphorylates nuclear factor of activated T cells (NFATs), a prominent regulator of cell differentiation and adaptation, leading to a decline in nuclear translocation of NFATs, resulting in reduced levels of nuclear NFATs^12–15^. In this study, continuous single-dose ISO reduced the nuclear levels of NFATc1/c2 while promoting the accumulation levels of NFATc3/c4 in the nucleus, suggesting that these special changes in NFATs participated in ISO-mediated alterations in myoblast differentiation/fusion, myotube size and myofiber specialization.

## Method

### C2C12 myoblast culture and differentiation induction

C2C12 myoblasts (purchased from the Cell Resource Center of Shanghai Academy of Life Sciences, Chinese Academy of Sciences) were inoculated in 75 cm^2^ culture dishes and cultured in proliferation medium (PM) containing 10% FBS (C0225, AusGenex Fetal Bovine Serum Excellent) plus high glucose DMEM (Gibco, USA, HG-DMEM) at 37 °C and 5% CO_2_. Cell culture continued under differentiation medium (DM) containing 2% horse serum (HS, BI 04-124-1A, Sigma, USA) plus HG-DMEM to induce C2C12 myoblast cell differentiation when the cell density reached approximately 75%. Traits of myotubes from myoblast differentiation were observed every day under a microscope^16^.

### Method of ISO administration in vitro

C2C12 myoblast cells cultured under DM at 75% confluence were transiently, intermittently or continuously stimulated by single-dose ISO (Sigma, USA). Briefly, transient ISO administration was performed by adding single-dose ISO only once on the first day of differentiation. Interval ISO administration was carried out by adding single-dose ISO in an alternate-day manner when DM was replaced. Continuous ISO administration was executed by adding single-dose ISO each day when DM was renewed. 10^−8^ M, 10^−7^ M, 10^−6^ M or 10^−5^ M ISO were used to observe the dosage effect on myoblast differentiation and fusion and myotube size for 6 days.

### Overexpression or knockdown of NFATs in vitro

Constructions of NFATc1/c2/c4 overexpression adenoviral vectors were prepared as previously described^17^. The gene accession numbers of overexpressing NFATc1/c2/c4 are NM_172390, NM_173091, and NM_004554, respectively. Constructions of NFATc3/c4 short hairpin RNA (shRNA) adenoviral vectors were prepared as previously described^17^. These overexpression adenoviral vectors containing Ad-NFATc1, Ad-NFATc2, Ad-shNFATc3 and Ad-shNFATc4 were obtained from Vigenebio. To confirm the role of NFATc3 or NFAtc4 in myoblast cells, Ad-shCtrl, Ad-shNFATc3, or Ad-shNFATc4 (1 × 10^9^ pfu) was added to the corresponding culture dishes one day before ISO treatment. Then, these cells were replaced with differentiation medium for further observation.

### Immunofluorescence staining

First, monoclonal and polyclonal antibodies, including MyoG (sc-12732, 1:150, Santa Cruz), MEF2C (#5030 s, 1:200, CST), and MyHC (sc-20641, 1:150, Santa Cruz), were added to each well in every group and then incubated for 12 h at 4 °C. The incubated cells were washed with PBS 3 times for 15 min and subsequently treated with the appropriate fluorescent dye-labeled secondary antibodies (Jackson Lab, 1:500, USA) at 25 °C for 2 h. The nuclei were stained with DAPI (Molecular Probes). The images for each group were photographed under a Nikon 80i fluorescence microscope^18^.

### Myoblast differentiation

After myoblast cells were treated under DM for the indicated time, the differentiated myoblast cells were stained for MyoG or MEF2C through the first polyclonal antibody MyoG (sc-12732, 1:150, Santa Cruz) or MEF2C (5030S, 1:400, CST) and appropriative TRITC-labeled secondary antibody (Jackson Lab, 1:500, USA). The nuclei were marked by DAPI staining. The numbers of single- or double-positive nuclei in a high-power field (HPF, 50 μm) were analyzed after double staining with MyoG/DAPI or MEF2C/DAPI. Images were evaluated by two people who did not know the results using ImageJ (Java) software (National Institutes of Health, USA). The percentage was calculated by the formula = MyoG- or MEF2C-positive nucleus numbers/DAPI-positive nucleus numbers.

To further distinguish the difference between differentiation and fusion, MyoG/DAPI- or MEF2C/DAPI-positive cells were divided into two types, and the corresponding cell numbers were evaluated. C2C12 myoblast cells with only 1–2 nuclei within a cellular structure were evaluated as analyzed by MyoG or MEF2C staining, indicating that MyoG + or MEF2C + cells were defined as differentiated cells without mutual fusion to myotubes. Myoblast cells with 3 and more than 3 nuclei in the structure of a cell were defined as myotubes. The numbers of double-positive nuclei in a high-power field (HPF, 50 μm) were analyzed after double staining with MyoG/DAPI or MEF2C/DAPI. Images were evaluated by two people who did not know the results using ImageJ (Java) software (National Institutes of Health, USA).

### Myoblast fusion and myotube morphology

The differentiated myoblast cells were stained for MyHC through the first polyclonal antibody MyHC (rabbit anti-mouse antibody, sc-20641, 1:150, Santa Cruz) and appropriate TRITC or FITC-labeled secondary antibody (Jackson Lab, 1:500, USA). C2C12 myoblast cells with only 1–2 nuclei within a cellular structure were evaluated as analyzed by MyHC staining, indicating that MyHC + cells were defined as differentiated cells without mutual fusion to myotubes. Myoblast cells with 3 and more than 3 nuclei in the structure of a cell were defined as myotubes. The nuclei were marked by DAPI staining.

To further analyze myotube size, the myotubes were divided into two types, and the myotube numbers, length and area were evaluated. One is a short myotube with 3 ~ 5 myoblast fusions; the other is a long myotube with more than 5 myoblast fusions. Morphology was assessed by myotube length, area (two types, less than 200 μm and more than 200 μm) and number of myotubes with myoblast fusion (3 ~ 5 nuclei or more than 5 nuclei) under high-power magnification^17,19^. Images were evaluated by two people who did not know the results using ImageJ (Java) software (National Institutes of Health, USA).

### Quantitative RT‒PCR

Using the SuperScript II cDNA kit (Invitrogen, Life Technologies), the total RNA extracted from C2C12 myoblast cells by TRIzol reagents (Invitrogen, Life Technologies) was transcribed into cDNA. Quantitative PCR was carried out by using SYBR green PCR master mix (Thermo Fisher Scientific, Applied Biosystems, CN) in a Real-Time PCR System (RotorGene 6000, Qiagen, Germany). The transcript levels of the gene of interest in each group were normalized to the GAPDH levels^20^. The primers used are listed in Table 1.

**Table 1:** The sequences of primers of qPCR.

Gene	Forward	Reverse
MyoG	5’-GAGACATCCCCCTATTTCTACCA-3’	5’-GCTCAGTCCGCTCATAGCC-3’
MyoD1	5’-CCACTCCGGGACATAGACTTG-3’	5’-AAAAGCGCAGGTCTGGTGAG-3’
MYH7	5’-CAAGCAGCAGTTGGATGAGCGACT-3’	5’-TCCTCCAGCTCCTCGATGCGT-3’
MYH2	5’-AGAGGACGACTGCAGACCGAAT-3’	5’-GAGTGAATGCTTGCTTCCCCCTTG-3’
MYH4	5’-ACGCTTGCACACAGAGTCAG-3’	5’-CTTGGACTCTTCCTCTAGCTGCC-3’
MYH1	5’-ACCAAGGAGGAGGAACAGCAGC-3’	5’-GAATGCCTGTTTGCCCCTGGAG-3’
hNFATc1	5’-AAGCACCAGCTTTCCAGTCC-3’	5’-TGCATAGCCATAGTGTTCTTCC-3’
hBFATc2	5’CGATTCGGAGAGCCGGATAG-3’	5’-TGGGACGGAGTGATCTCGAT-3’
mNFATc3	5’-CTGTGCAAACCCCACCTC-3’	5’-GCCCAGAAATCGGTGAAC-3’
mNFATc4	5’-TACAGCAACAAGCGGGTGTC-3’	5’-CGGAGAGATGAGTCTGGTAGGG-3’
GAPDH	5’-ATGACTCCACTCACGGCAAA-3’	5’-ATGATGACCCTTTTGGCTCC-3’

### Western blot

C2C12 myoblast cells in the tubes were placed on ice and homogenized within 0.1% Tween-20 homogenization buffer with the addition of protease inhibitors. This was followed by the separation and collection of nuclear and cytosolic proteins from each group by using NE-PER Nuclear and Cytoplasmic Extraction Reagents according to the manufacturer’s instructions (78,835, Thermo Fisher Scientific, USA). After electrophoresis in a 7 or 10% SDS‒PAGE gel, 20 µg of protein from each group was transferred onto a PVDF membrane (Millipore). Then, the membrane was blocked with 5% nonfat milk. Subsequently, primary antibodies against α-Tubulin (T9026, 1:5000, Sigma), LaminB1 (ab16048, 1:1000, Abcam), GAPDH (Ap0066, 1:10,000, Bioworld), Histone H3 (ab6002, 1:500, Abcam), His-Tag (ab9136, 1:1000, Abcam), NFATc1 (ab2796, 1:500, Abcam), NFATc2 (ab2722, 1:500, Abcam), NFATc3 (ab83832, 1:500, Abcam), NFATc4 (SAB4501982, 1:1000, Sigma) and MyHC (sc-20641, sc-376157, 1:500, Santa Cruz) were added to the incubation solution and incubated with the membrane overnight at 4 °C. Finally, the corresponding horseradish peroxidase (HRP)-conjugated secondary antibodies (anti-rabbit IgG, anti-goat IgG, 1:10,000; Santa Cruz) were added to the incubation solution for 90 min. The changes in protein expression were developed by the chemiluminescence method, and the gray values were analyzed for semi-quantitative analysis by using ImageJ software^21^.

### Statistical analysis

IBM SPSS statistics software (version 22, 32-bit edition) was used for for statistical analysis. https://www.ibm.com/cn-zh/products/spss-statistics. Data from quantitative and semi-quantitative analyses are presented as the mean ± SD. The paired or unpaired Student’s t test was used to determine statistical significance between two groups. Comparison of results among more than three experimental groups should be made to specify their differences by one-way ANOVA. *P* < 0.05 was considered meaningful.

IBM SPSS statistics, version 22, 32-bit edition.

## Results

### Continuous single-dose ISO most obviously hampered C2C12 myoblast differentiation and fusion

**Figure 1:**
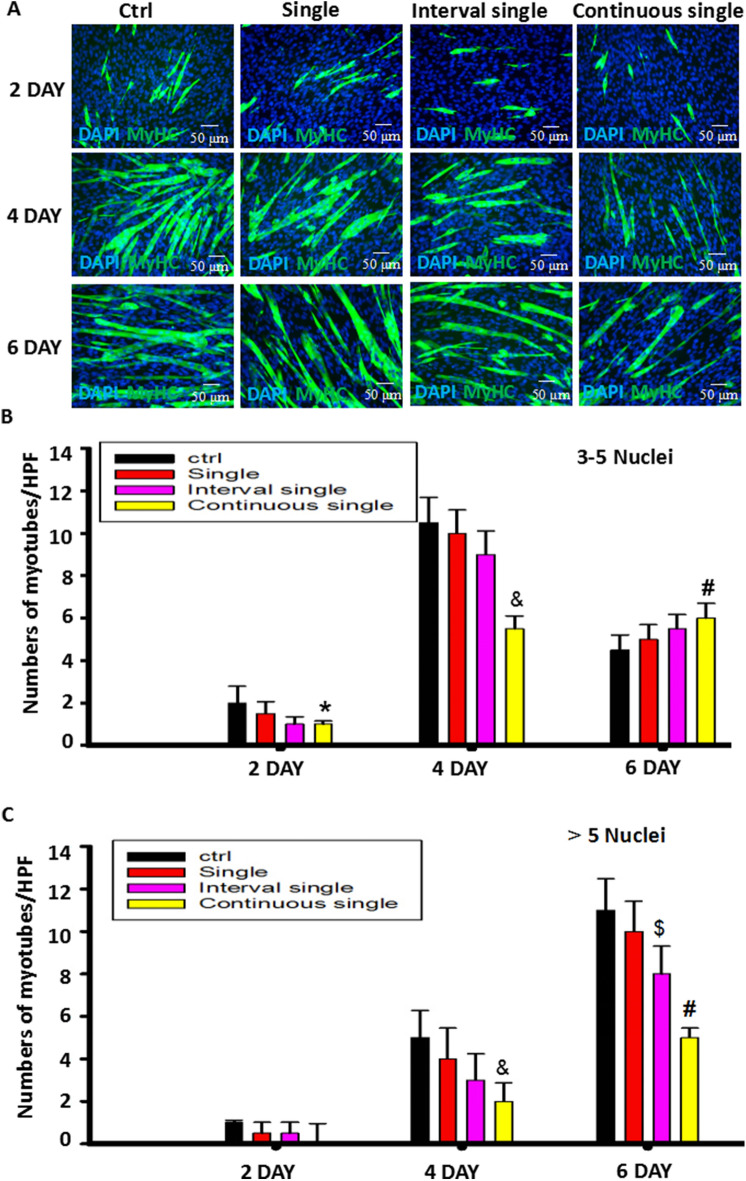
Continuous stimulation with ISO most obviously inhibited C2C12 cell differentiation and myoblast fusion. (**A**) Typical image of MyHC staining in differentiated C2C12 myoblast cells exposed to different delivery methods of ISO. (**B**) A quantitative assay for the number of MyHC + myotubes with 3–5 nuclei was performed 2, 4 and 6 days after myoblast differentiation following stimulation with ISO delivered with single, interval single or continuous single doses. n = 3, ^*#*^*P* < 0.05 versus Ctrl. (**C**) A quantitative assay for the number of MyHC + myotubes with more than 5 nuclei was performed 2, 4 and 6 days after myoblast differentiation following stimulation with ISO delivered with single, interval single or continuous single doses. n = 3, & *P* < 0.05 versus Ctrl; ^*#*^*P* < 0.05 versus Ctrl, single or interval single group; ^*$*^*P* < 0.05 versus Ctrl.

To confirm the distinct role of ISO on myoblast differentiation and fusion, we administered ISO at different frequencies, including once single-dose, interval single-dose or continuous single-dose, and detected the morphological changes of myotubes by MyHC immunostaining. As shown in Fig. 1, in differentiation medium containing 2% HS-DMEM, C2C12 myoblast cells time-dependently differentiated into mature muscle cells and formed myotubes characterized by MyHC-positive staining. Regardless of single-dose, interval single-dose or continuous single-dose delivery, there was no difference in myotube numbers with either 3–5 nuclei or more than 5 nuclei (5^+^) on day 2. However, on day 4, continuous single-dose ISO not only decreased the numbers of myotubes with 3–5 nuclei but also reduced the numbers of myotubes with 5^+^ nuclei, suggesting that continuous single-dose ISO hindered C2C12 myoblast differentiation and postponed myoblast fusion. Furthermore, on the fourth and sixth days of differentiation, there was almost a 50% inhibition ratio of myotube numbers with 5^+^ nuclei in response to continuous single-dose ISO compared to the one single-dose or interval single-dose ISO, indicating that continuous single ISO could stably inhibit myoblast fusion.

**Figure 2:**
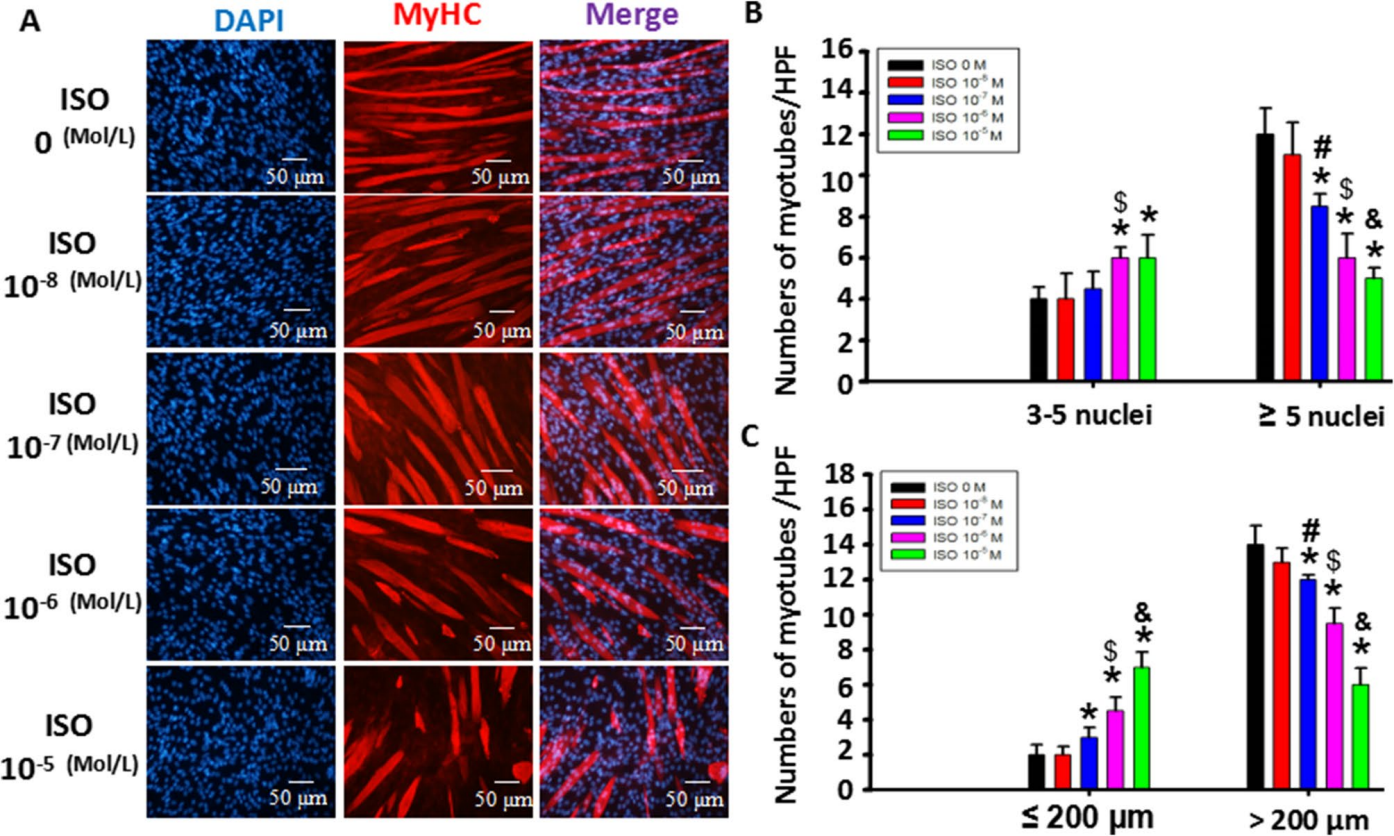
Continuous ISO stimulation dose-dependently altered NFATc1 and NFATc2 signaling. (**A**) Typical image of MyHC staining in differentiated C2C12 myoblast cells exposed to continuous single-dose 10^−5^ M ISO. (**B**) A quantitative assay for the number of MyHC + myotubes with 3–5 or more than 5 nuclei was performed six days after myoblast differentiation following stimulation with ISO delivered with a continuous single dose. n = 3, *P < 0.05 versus Ctrl; ^*$*^*P* < 0.05 versus 10^−8^ M ISO or 10^−7^ M ISO; ^*#*^*P* < 0.05 versus 10^−8^ M ISO; ^*&*^*P* < 0.05 versus 10^−8^ M ISO or 10^−7^ M ISO. (**C**) Quantitative assays for the number of MyHC + myotubes with lengths of less than 200 μm or more than 200 μm were performed six days after myoblast differentiation following stimulation with ISO delivered with a continuous single dose. n = 3, *P < 0.05 versus Ctrl; ^*$*^*P* < 0.05 versus 10^−8^ M ISO or 10^−7^ M ISO; ^*#*^*P* < 0.05 versus 10^−8^ M ISO; ^*&*^*P* < 0.05 versus 10^−8^ M ISO, 10^−7^ Mor1 0^−6^ M ISO.

To further determine the dose-dependent effect of ISO with single-dose stimulation on C2C12 myoblast differentiation and fusion, 10^−8^, 10^−7^, 10^−6^ or 10^−5^ mol/L ISO was used to treat the cells once every day. As shown in Fig. 2, compared to the normal differentiation group, the number of MyHC-positive myotubes with either 3–5 or 5^+^ nuclei was evidently decreased with continuous single-dose ISO stimulation in a dose-dependent manner, especially 10^−5^ mol/L ISO, reducing the number of 5^+^ nuclei myotubes by nearly two-thirds, indicating that continuous single-dose ISO inhibited C2C12 myoblast differentiation and fusion. Furthermore, in response to continuous single-dose ISO, the numbers of less than 200 μm myotubes were dose-dependently increased, while the numbers of more than 200 μm (200^+^) myotubes were decreased, especially 10^−5^ mol/L ISO, diminishing the numbers of 200^+^ myotubes by nearly 50% compared to the normal differentiation group. Meanwhile, it increased the numbers of less than 200 μm myotubes by 3.5 times as much as the control group, indicating that continuous single-dose ISO substantially shortened the length of myotubes. In summary, continuous single-dose ISO diminished myotube size by hampering C2C12 myoblast differentiation and fusion.

### Continuous ISO stimulation dose-dependently altered NFATc1 and NFATc2 signaling

**Figure 3:**
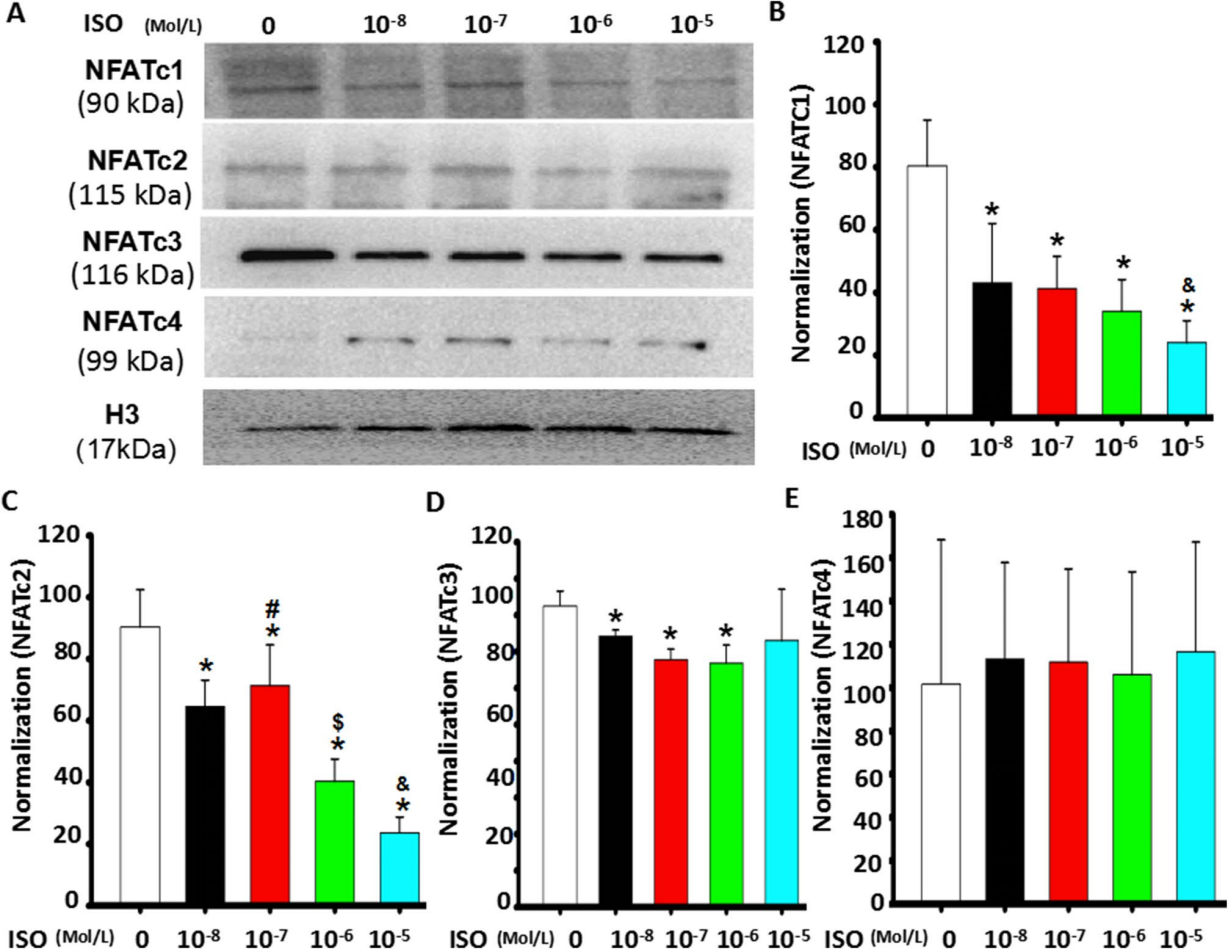
Continuous ISO stimulation dose-dependently altered NFATc1 and NFATc2 signaling. (**A**) Continuous ISO stimulation dose-dependently reduced nuclear levels of NFATc1 and NFATc2 while slightly increasing nuclear NFATc4 levels, as determined by western blot, five days after myoblast differentiation following stimulation with ISO delivered with a continuous single dose. (**B**–**E**) Semi-quantitative assay from Fig. 3A. n = 3, *P < 0.05 versus Ctrl; ^*#*^*P* < 0.05 versus 10^−8^ M ISO; ^*$*^*P* < 0.05 versus 10^−8^ M ISO or 10^−7^ M ISO. ^*&*^*P* < 0.05 versus 10^−8^ M ISO, 10^−7^ M ISO or 10^−5^ M ISO.

To further determine whether the dose-dependent effect of ISO with single-dose stimulation on C2C12 myoblast differentiation and fusion involved in NFAT signaling, different doses of ISO, including 10^−8^, 10^−7^, 10^−6^ or 10^−5^ mol/L, were continuously added to the medium containing 2% HS-DMEM. As shown in Fig. 3, continuous single-dose ISO dramatically reduced NFATc1 and NFATc2 in differentiating C2C12 myoblast cells in a dose-dependent manner on the 5th day of differentiation, particularly 10^−5^ mol/L ISO. In contrast, it slightly increased the levels of NFATc3 and NFATc4. These results demonstrated that NFATc1 and NFATc2 signaling were involved in the regulation of myoblast differentiation and fusion.

### Continuous ISO stimulation time-dependently altered NFATc1 and NFATc2 signaling

**Figure 4:**
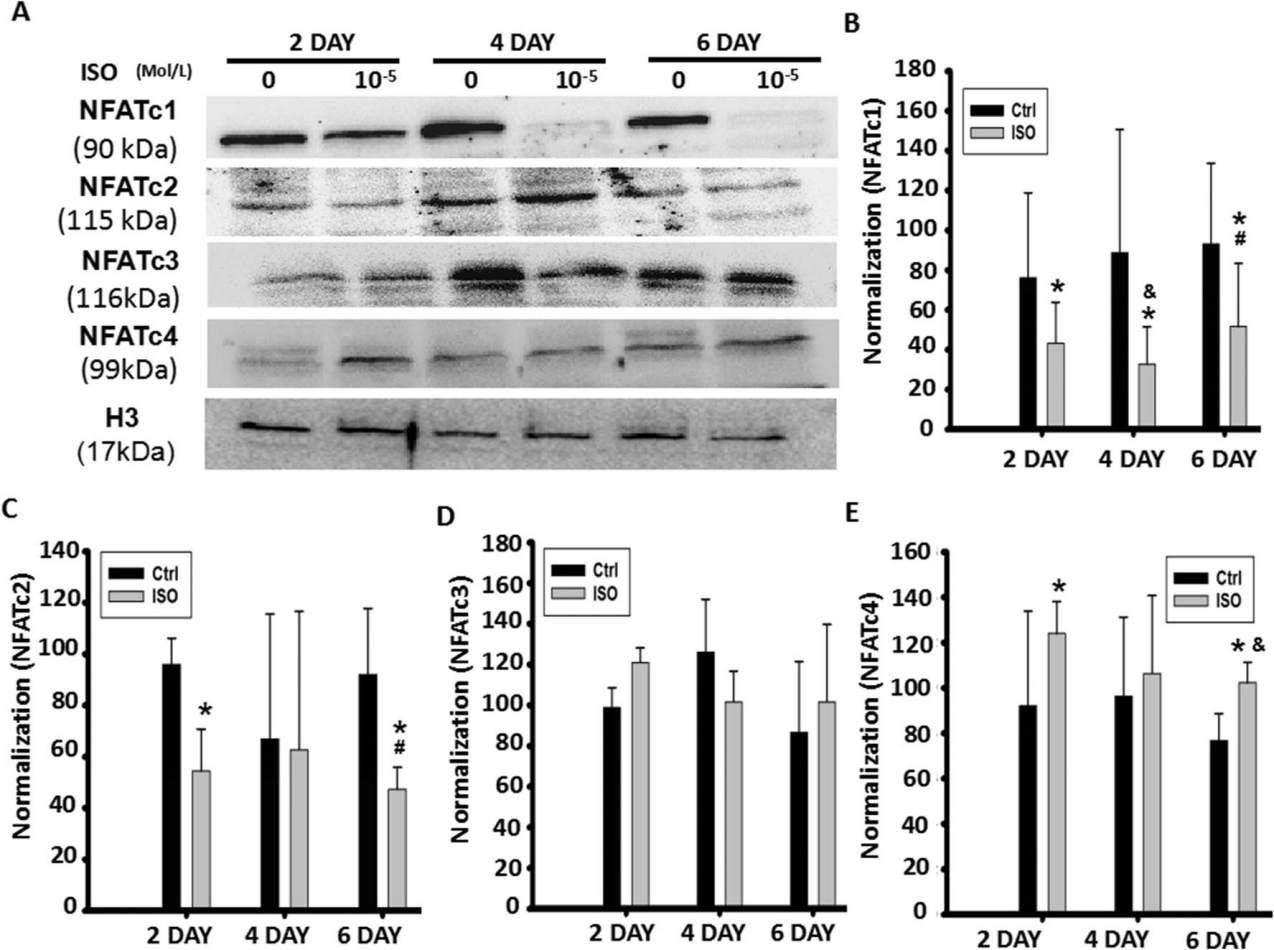
Continuous ISO stimulation time-dependently altered NFATc1 and NFATc2 signaling. (**A**) Continuous ISO stimulation reduced nuclear levels of NFATc1 and NFATc2 while slightly increasing nuclear levels of NFATc3 and NFATc4, as determined by western blot, 2, 4 and 6 days after myoblast differentiation following the stimulation of ISO delivered with a continuous single dose. (**B**–**E**) Semi-quantitative assay from Fig. 3A. Three independently repeated experiments were performed. n = 3, *P < 0.05 versus Ctrl at the indicated times*; *^*&*^*P* < 0.05 versus the 10^−5^ M ISO group on the second day of myoblast differentiation; ^*#*^*P* < 0.05 versus the 10^−5^ M ISO group on the 4th day of myoblast differentiation.

To further determine the time-dependent involvement of NFAT signaling in C2C12 myoblast differentiation and fusion, western blotting was used to evaluate changes in protein levels at different times. As shown in Fig. 4A–E, in the normal differentiation group, there was a gradual increase in NFAT levels within 6 days. On day 4, the protein levels of NFATc1 ~ c4 were evidently increased compared to the levels on day 2 (Fig. 4A–E). However, the protein levels on day 6 of differentiation were markedly decreased compared to those on day 4. These results indicated the dynamics of NFAT expression involved in myoblast differentiation and fusion.

Following the stimulation of continuous single-dose ISO with 10^−5^ mol/L, we found that NFATc1 levels in myoblast cells continuously decreased, while NFATc3 and NFATc4 levels increased. Similar to the normal differentiation group, NFATc2 presented an alternative pattern where there was a decrease and then an increase in levels during myoblast differentiation when stimulated with ISO. Regardless of a decrease or an increase, ISO treatment significantly influenced NFATc2 levels more than the control treatment (Fig. 4A–E). These typical changes indicated that NFAT signaling could participate in the process of myoblast differentiation and fusion mediated by ISO.

### NFAT signaling is involved in the regulation of myoblast differentiation/fusion and myotube size mediated by ISO

**Figure 5:**
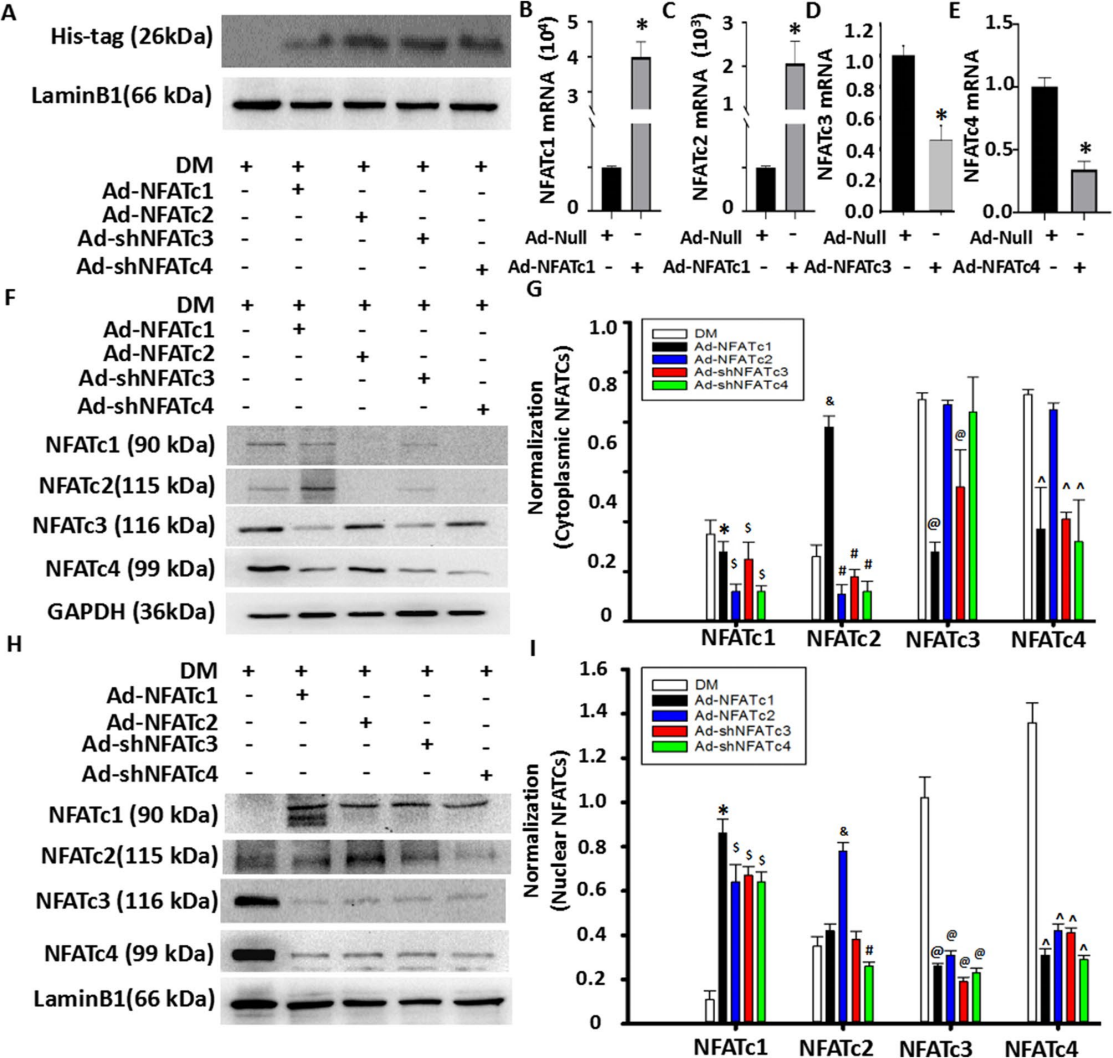
Transfection efficiency of NFAT overexpression or knockdown in C2C12 myoblast cells. (**A**) Western blot showed that the adenovirus vector was successfully transferred into myoblasts by detecting Flag-His after transfection with 100 optimal multiplications of infection (MOI) for the indicated adenovirus expression vectors. (**B**–**E**) Quantitative analysis of the transfection efficiency of these specific adenoviruses into C2C12 myoblast cells was determined by qPCR after myoblast cells were transfected with adenovirus-mediated overexpression of NFATc1 and NFATc2 or knockdown of NFATc3 and NFATc4 by shRNA (100 MOI) for 72 h. n = 3, *P < 0.05 versus Ad-Null. (**F**) Cytoplasmic proteins were evaluated by using western blotting. (**G**) Semi-quantitative assay from Fig. 5C. Three independently repeated experiments were performed. n = 3, ^*^P < 0.05 versus DM;^&^*P* < 0.05 versus 10^−5^ M ISO group; ^#^*P* < 0.05 versus 10^−5^ M ISO group; ^$^*P* < 0.05 versus 10^−5^ M ISO + Ad-shNFATc3 group. (**H**–**I**) Western blot and semi-quantitative assays were used to evaluate nuclear proteins in these adenovirus-treated cells. n = 3, ^*^P < 0.05 versus DM;^&^*P* < 0.05 versus 10^−5^ M ISO group; ^#^*P* < 0.05 versus 10^−5^ M ISO group; ^$^*P* < 0.05 versus 10^−5^ M ISO + Ad-shNFATc3 group;^^^*P* < 0.05 versus DM.

To determine the relationship between NFATs and ISO-mediated myoblast differentiation and fusion inhibition, adenovirus-mediated overexpression of NFATc1 and NFATc2 or knockdown of NFATc3 and NFATc4 by shRNA were used to observe the ISO-mediated effect on myoblast differentiation and fusion inhibition. First, His-tag, as an adenovirus vector-carried reporter gene, was detected, indicating that the adenovirus vector was successfully transfected with the results of the marked expression in myoblasts (Fig. 5A). Second, overexpressed NFATc1 or NFATc2 in myoblasts showed a 3–fourfold increase in mRNA levels (Fig. 5B,C), implying that NFATc1/c2 was successfully overexpressed in myoblasts. Knocking down NFATc3 and NFATc4 in myoblasts obliviously decreased the 70% mRNA levels (Fig. 5D,E), indicating that NFATc3/c4 were successfully knocked down in myoblasts. Finally, to confirm whether overexpression or knockdown of NFATs affects the expression and cytoplasm/nucleus distribution of other NFATs, we used western blot analysis. We found that overexpression of NFATc1 dramatically increased the nuclear NFATc1 and cytoplasmic NFATc2 levels while reducing the nuclear and cytoplasmic NFATc3/c4 and cytoplasmic NFATc1 levels (Fig. 5F,G). The difference was that overexpression of NFATc2 did not alter the cytoplasmic levels of NFATc3/c4 but obviously increased the nuclear NFATc1/c2 levels while lessening the NFATc3/c4 levels in nuclei (Fig. 5H,I). Of interest, while knockdown of NFATc3 by shRNA caused a decrease in nuclear and cytoplasmic NFATc3 levels, the nuclear levels of NFATc1/c2 were increased, especially NFATc1. Knockdown of NFATc4 by shRNA also increased the levels of nuclear NFATc1 but reduced the levels of nuclear NFATc2/c3 and cytoplasmic NFATc1/c2, accompanied by a decrease in nuclear and cytoplasmic NFATc4 levels (Fig. 5F–I). These results indicated that NFAT interactions could be involved in the changes in myoblast differentiation and fusion induced by ISO.

**Figure 6:**
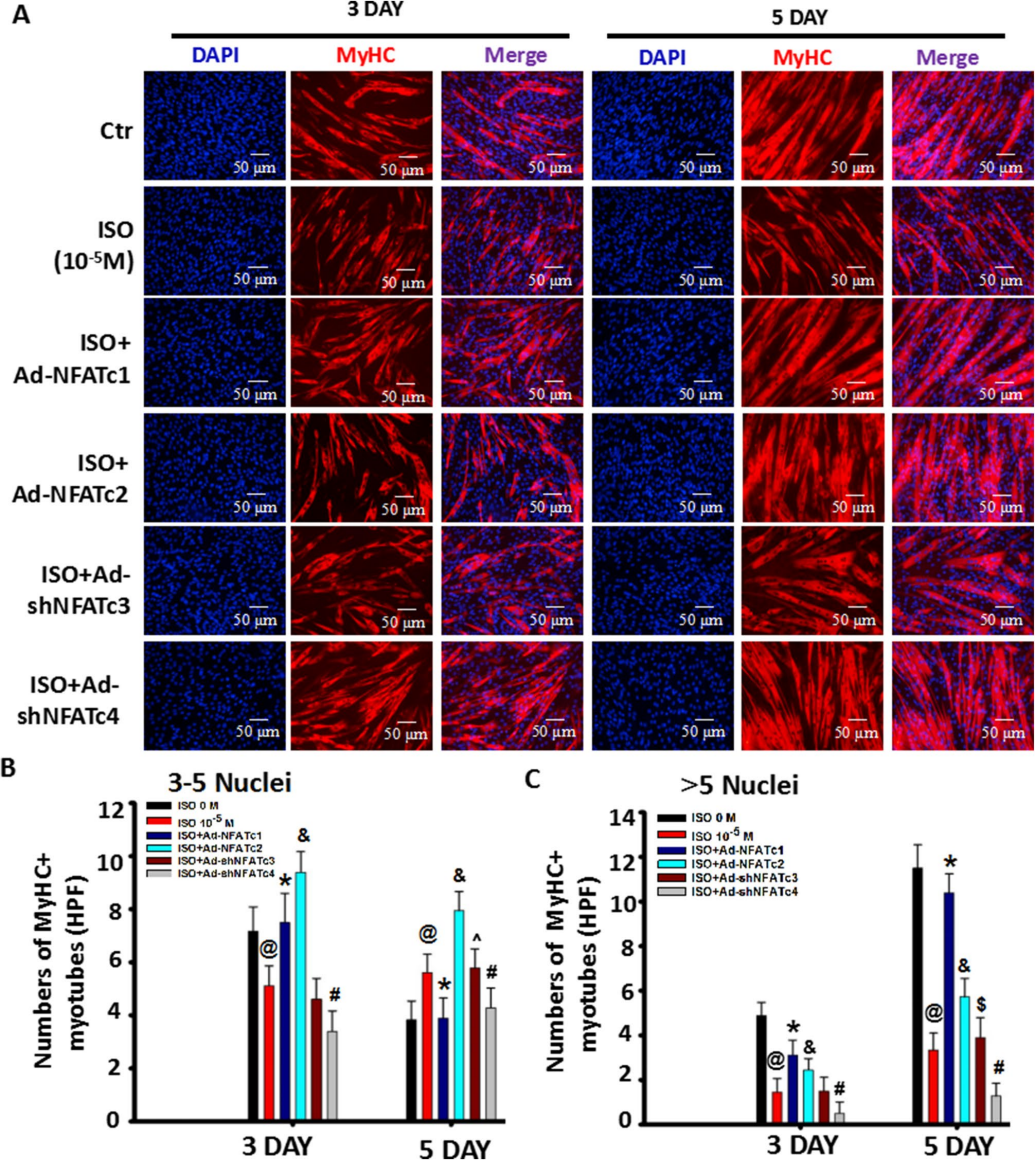
NFAT signaling is involved in the regulation of myotube size mediated by ISO. (A) Myoblast cells were stimulated with continuous single-dose ISO in differentiation medium for three and five days and stained with MyHC 24 h after transfection with adenovirus-mediated overexpression of NFATc1 (Ad-NFATc1) and NFATc2 (Ad-NFATc2) or knockdown of NFATc3 (Ad-shNFATc3) and NFATc4 by shRNA (Ad-shNFATc4) at 100 MOI. Three independently repeated experiments were performed. Then, a quantitative assay for the number of MyHC + cells with 1–2 nuclei was performed, as shown in (**A**). n = 3, ^*@*^*P* < 0.05 versus Ctrl;^*&*^*P* < 0.05 versus 0 M ISO, 10^−5^ M ISO, ISO + Ad-NFATc1, ISO + Ad-NFATc12 or ISO + AdshNFATc4 group; ^*#*^*P* < 0.05 versus all groups; *P < 0.05 versus all groups. (**B**) Quantitative assay for the number of MyHC + myotubes with 3–5 nuclei from Fig. 6. n = 3, ^*@*^*P* < 0.05 versus Ctrl; *P < 0.05 versus ISO group;^*&*^*P* < 0.05 versus 0 M ISO, 10^−5^ M ISO or ISO + Ad-NFATc1 group; ^*#*^*P* < 0.05 versus ISO group or ISO + AdshNFATc3 group; ^ *P* > 0.05 versus ISO group. (**C**) Quantitative assay for the number of MyHC + myotubes with more than 5 nuclei from (**A**). n = 3, ^*@*^*P* < 0.05 versus Ctrl; *P < 0.05 versus ISO group;^*&*^*P* < 0.05 versus 0 M ISO, 10^−5^ M ISO or ISO + Ad-NFATc1 group; ^*#*^*P* < 0.05 versus ISO group or ISO + AdshNFATc3 group; ^*$*^*P* > 0.05 versus ISO group. Three independently repeated experiments were performed.

**Figure 7:**
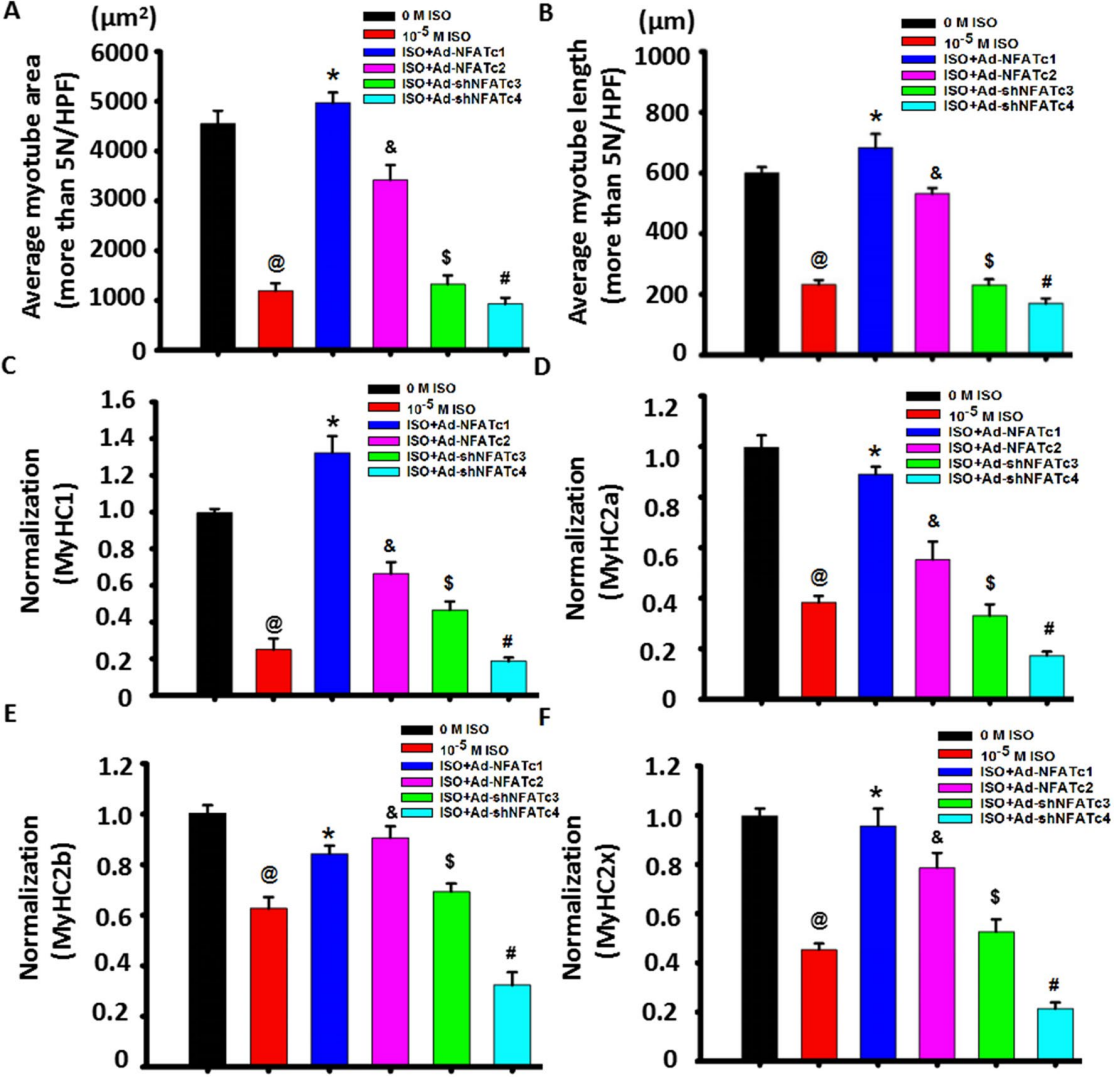
NFAT signaling is involved in the regulation of myotube types mediated by ISO. (A) Myoblast cells were stimulated with continuous single-dose ISO under DM for five days and stained with MyHC 24 h after the cells were transfected with Ad-NFATc1, Ad-NFATc2, Ad-shNFATc3 or Ad-shNFATc4 at 100 MOI. The average myotube area of MyHC + myotubes with 5 + nuclei is shown in Fig. 6, as detected by MyHC staining. n = 3, ^*@*^*P* < 0.05 versus Ctrl; *P < 0.05 versus ISO group; ^*&*^*P* < 0.05 versus ISO group or ISO + Ad-NFATc1 group; ^*#*^*P* > 0.05 versus ISO group; ^*$*^*P* > 0.05 versus ISO group. N: nuclei. (**B**) Quantitative assay for average myotube length of MyHC + myotubes with 5 + nuclei from Fig. 6. n = 3, ^*@*^*P* < 0.05 versus Ctrl; *P < 0.05 versus ISO group;^*&*^*P* < 0.05 versus ISO group or ISO + Ad-NFATc1 group; ^*#*^*P* > 0.05 versus ISO group; ^*$*^*P* > 0.05 versus ISO group. (**C**–**F**) Myoblast cells were transfected with the indicated adenovirus for 24 h prior to stimulation with continuous single-dose ISO for six days. Then, real-time PCR was used to evaluate MyHC1, MyHC2a, MyHC2b and MyHC2x mRNA expression in differentiated myoblast cells. n = 3, ^*@*^*P* < 0.05 versus Ctrl; *P < 0.05 versus ISO group;^*&*^*P* < 0.05 versus ISO group or ISO + Ad-NFATc1 group; ^*$*^*P* > 0.05 versus ISO group; ^*#*^*P* > 0.05 versus ISO group. Three independently repeated experiments were performed.

As per their respective functions, the overexpression of NFATc1/c2 abolished the suppressive effects of ISO on myoblast differentiation and myoblast fusion (Fig. 6). Moreover, overexpression of NFATc1 resulted in a more potent effect than overexpression of NFATc2 in restoring myoblast differentiation/fusion characterized by 5^+^ nuclei myotubes (Fig. 6A–C). Compared with the partial recovery of the ISO-mediated inhibition effect by overexpression of NFATc2, forced NFATc1 expression restored myotube length and area relative to the sizes in the normal differentiation group (Figs. 6A–C, 7A–B).

Of interest, Ad-shNFATc3 did not alter the ISO-mediated inhibition of myoblast differentiation/fusion compared with Ad-shNFATc4, worsening it. Although knockdown of NFATc4 by shRNA increased MyHC-positive cells with 1–2 nuclei in ISO-treated myoblast cells, it decreased the number of both 3–5 nuclei and 5^+^ nuclei myotubes (Fig. 6A–C, Supplemental Fig. 1A), leading to a smaller myotube length and area (Figs. 1B and 7A–B), indicating that it did not change myoblast differentiation but did change myoblast fusion, which was associated with the decrease in NFATc2 and increase in NFATc1 induced by knocking down NFATc4 (Fig. 5E–H). Meanwhile, overexpression of NFATc4 partially restored the inhibitory role of ISO in myoblast fusion (Supplemental Fig. 2). Unlike knocking down NFATc4, knocking down NFATc3 did not alter the inhibitory role of ISO on myotube length and area, which was related to the increase in NFATc1/c2 induced by knocking down NFATc3 (Fig. 5E–H).

Taken together, NFAT interactions could be involved in the inhibitory effect of ISO on myoblast differentiation/fusion and myotube length/size.

### NFAT signaling is involved in alterations in myofiber marker genes mediated by ISO

Previous studies have reported on two types of muscle fibers, including slow (slim-long) and fast (thick-short) myofibers. MyHC1 encoded by the Myh7 gene forms the former, and MyHC2a encoded by the Myh2 gene, MyHC2b encoded by the Myh4 gene or MyHC2X encoded by the Myh1 gene the latter^22,23^. Corresponding with decreased myotube formation following treatment with continuous single-dose ISO (Figs. 1, 2), the expression levels of Myh7, Myh4, Myh2 and Myh1 were significantly reduced (Fig. 7C–F). Moreover, there was a significant reduction in the expression of MyHC1, MyHC2a and MyHC2X compared with MyHC2b (Fig. 7C–F). Thus, continuous single-dose ISO hindered the expression of Myh7, Myh4, Myh2 and Myh1. Consistent with the results of myotube size by NFATc1 and NFATc2 overexpression or NFATc3 and NFATc4 knockdown by shRNA in ISO-treated myoblast cells (as shown in Fig. 7C–F), NFATc1 overexpression almost completely reversed the inhibitory effects of ISO on Myh7, Myh4 and Myh2 and Myh1 expression. NFATc2 overexpression partially recovered the expression of these genes in ISO-treated cells. NFATc3 knockdown did not alter the expression of yh1, Myh2 and Myh4 but partially reversed Myh7 expression in ISO-stimulated myoblast cells, while NFATc4 knockdown further reduced their expression. Therefore, continuous single-dose ISO could increase the number of fast and slow myofibers characterized by the size of myotubes in morphology by targeting NFAT signaling.

### NFATs are involved in the inhibitory effect of ISO on myoblast differentiation/fusion through coordination with MyoG and MEF2C

**Figure 8:**
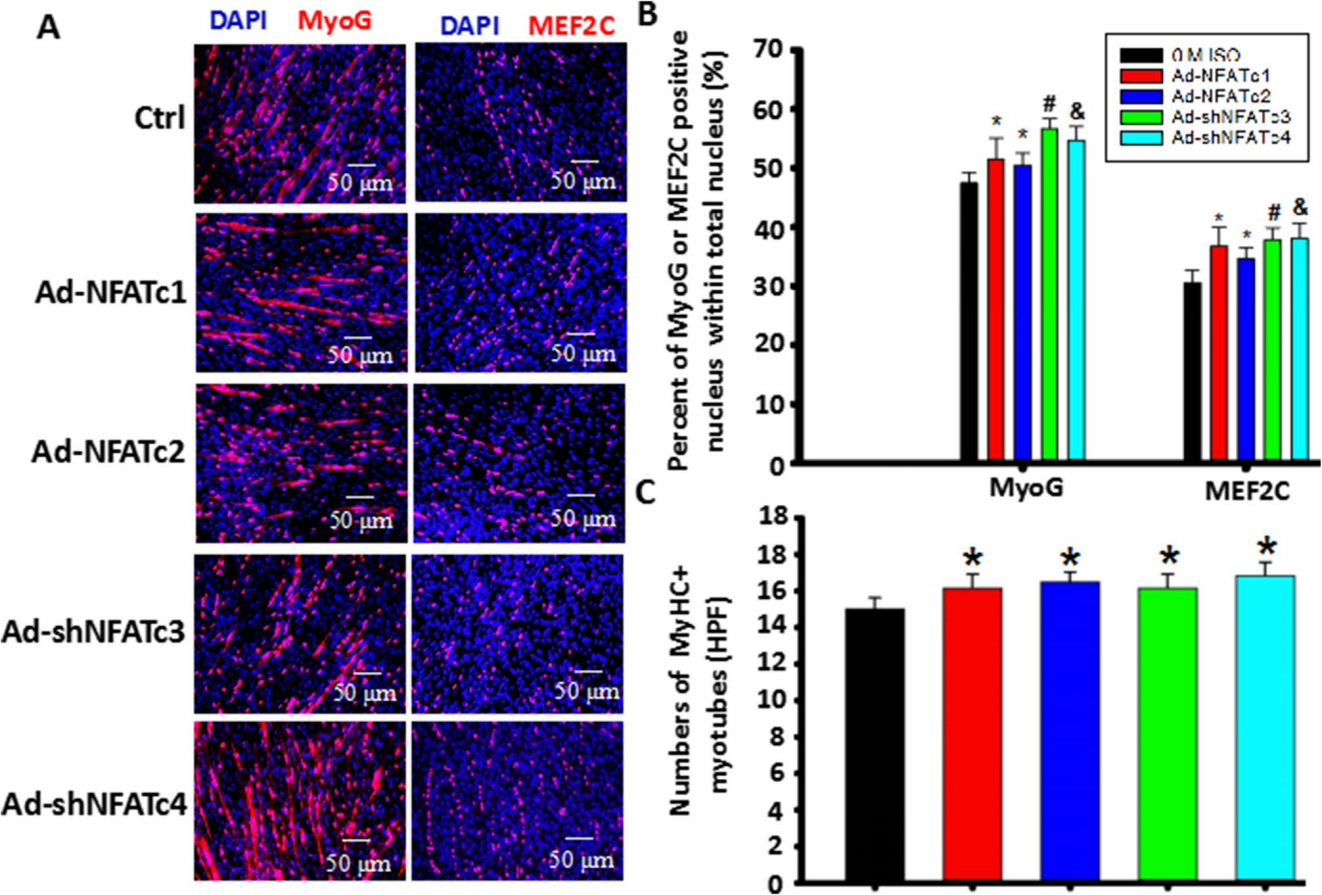
MyoG and MEF2C are involved in C2C12 myoblast differentiation mediated by the NFAT signaling pathway. Myoblast cells were cultured under DM for six days and then stained with MyoG or MEF2C 24 h after the cells were transfected with Ad-NFATc1, Ad-NFATc2, Ad-shNFATc3 or Ad-shNFATc4 at 100 MOI. Blue: DAPI-stained nuclei; Red: MEF2C or MyoG. (**B**) Quantitative assay for the number of MyoG or MEF2C + nuclei in the cells with less than 3 nuclei from 8A. n = 3, ^*@*^*P* < 0.05 versus Ctrl; *P < 0.05 versus ISO group;^*&*^*P* < 0.05 versus ISO + Ad-NFATc1 group; ^*$*^*P* > 0.05 versus ISO + Ad-NFATc1 group; ^*#*^*P* < 0.05 versus ISO + Ad-NFATc1 group. (**C**) Quantitative assay for the number of MEF2C + nuclei in the cells with more than 3 nuclei from 8A. n = 3, ^*@*^*P* < 0.05 versus Ctrl; *P < 0.05 versus ISO group;^*&*^*P* < 0.05 versus ISO group; ^*$*^*P* > 0.05 versus ISO group; ^*#*^*P* < 0.05 versus ISO + Ad-shNFATc3, ISO + Ad-NFATc1 or ISO + Ad-NFATc2 group.

Since MyoG and MEF2C play a crucial role in the initiation and subsequent processes of myoblast cell differentiation^24^, we used MyoG and MEF2C staining to confirm the relationship between NFATs and ISO-mediated myoblast differentiation. First, we observed whether specific overexpression or knockdown of NFATs affected the nuclear levels of MyoG and MEF2C. We found that the ratio of MyoG- or MEF2C-positive nuclei within the total nucleus was increased in these adenovirus treatment groups, resulting in increased numbers of myotubes (Fig. 8A–C). The percentage of MyoG^+^ nuclei was higher than that of MEF2C + nuclei in the differentiating C2C12 myoblast cells treated with these adenoviruses (Fig. 8A–C), indicating that MyoG/MEF2C are involved in myoblast differentiation and fusion mediated by NFATs.

**Figure 9:**
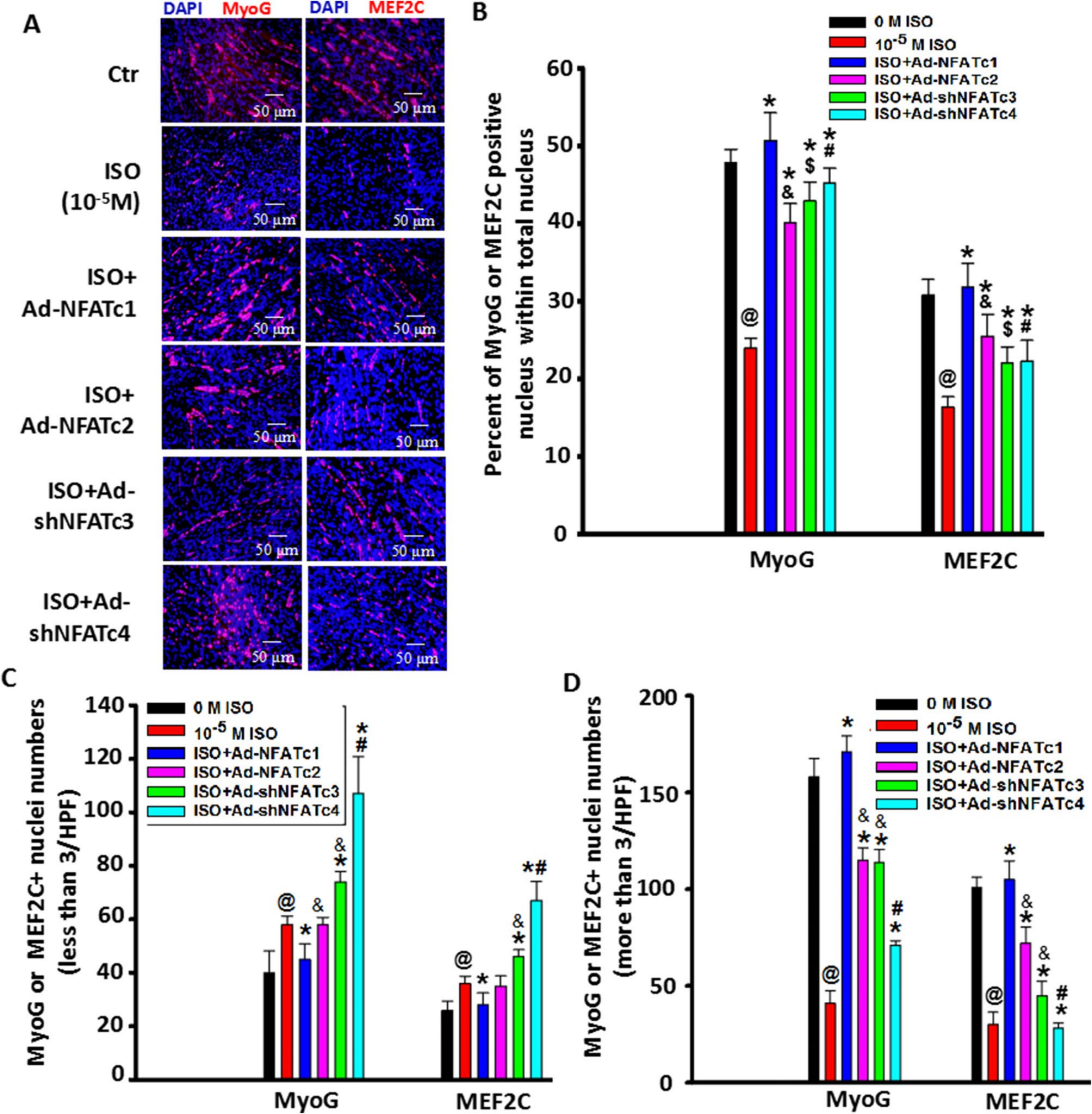
NFATs are involved in inhibiting C2C12 myoblast cell differentiation/fusion by ISO through coordination with MEF2C. Myoblast cells were stimulated by continuous single-dose ISO under DM for six days and then stained with MyoG or MEF2C 24 h after the cells were transfected with Ad-NFATc1, Ad-NFATc2, Ad-shNFATc3 or Ad-shNFATc4 at 100 MOI. Blue: DAPI-stained nuclei; Red: MEF2C. (**B**) The percentage of MyoG- or MEF2C-positive nuclei within the total nucleus was analyzed. n = 3, ^*@*^*P* < 0.05 versus Ctrl; *P < 0.05 versus ISO group; ^*&*^*P* < 0.05 versus ISO + Ad-NFATc1 group; ^*$*^*P* > 0.05 versus ISO + Ad-NFATc1 group; ^*#*^*P* < 0.05 versus ISO + Ad-NFATc1 group. (**C**) Quantitative assay for the number of MyoG or MEF2C + nuclei in the cells with fewer than 3 nuclei from 9A. n = 3, ^*@*^*P* < 0.05 versus Ctrl; *P < 0.05 versus ISO group;^*&*^*P* < 0.05 versus ISO + Ad-NFATc1 group; *#P* < 0.05 versus ISO + Ad-NFATc1 group. (**D**) Quantitative assay for the number of MyoG or MEF2C + nuclei in the cells with more than 3 nuclei from 9A. n = 3, ^*@*^*P* < 0.05 versus Ctrl; *P < 0.05 versus ISO group;^*&*^*P* < 0.05 versus ISO + Ad-NFATc1 group; ^*#*^*P* < 0.05 versus ISO + Ad-NFATc1, ISO + Ad-NFATc2 or ISO + Ad-shNFATc3 group.

Subsequently, with continuous single-dose ISO treatment, the percentage of MyoG- and MEF2C-positive nuclei in C2C12 myoblast cells was markedly reduced (Fig. 9A,B). The percentage of MEF2C^+^ nuclei was lower than that of MyoG + nuclei in ISO-treated C2C12 myoblast cells, indicating that ISO inhibited the initiation and subsequent processes of myoblast differentiation through MyoG and MEF2C, especially in MEF2C. Combined with the results that ISO did not significantly hamper the initial differentiation of myoblasts shown in Fig. 1, ISO mainly inhibited the anaphase of myoblast differentiation through MEF2C. More importantly, overexpressing NFATc1/c2 or knocking down NFATc3/c4 almost completely restored the number of MyoG- and MEF2C-positive nuclei in ISO-treated myoblasts (Fig. 9A,B). Moreover, among these four adenoviruses, overexpressing NFATc1 restored the positive number of MyoG and MEF2C most strongly close to the normal level, leading to the recovery of myoblast differentiation. These results demonstrated that ISO inhibited myoblast differentiation through the NFATs-MyoG/MEF2C signaling pathway.

Myotube formation results from the fusion of differentiated myoblasts, characterized by more than three (3^+^) nuclei in the structure of a cell^17,19^. We found that continuous single-dose ISO markedly decreased the numbers of MyoG- and MEF2C-positive nuclei in 3^+^ myotubes, especially in MEF2C myotubes. Meanwhile, overexpression of NFATc1 almost completely restored the number of MyoG- or MEF2C-positive nuclei in 3^+^ myotubes in ISO-treated myoblasts, close to normal levels (Fig. 9A–D). However, overexpression of NFATc2 in ISO-treated groups only partially recovered these changes. Similarly, knocking down NFATc3 partially recovered MyoG- or MEF2C-positive nuclei numbers in 3^+^ myotubes exposed to ISO. Interestingly, NFATc4 knockdown did not alter the reduced trends of MEF2C-positive nuclei in 3^+^ myotubes induced by ISO but partially recovered MyoG-positive numbers in 3^+^ myotubes (Fig. 9A–D). These results indicated that NFATs, NFATc1 in particular, participated in the suppressive role of continuous single-dose ISO in C2C12 myoblast fusion in coordination with MyoG and MEF2C.

## Discussion

**Figure 10:**
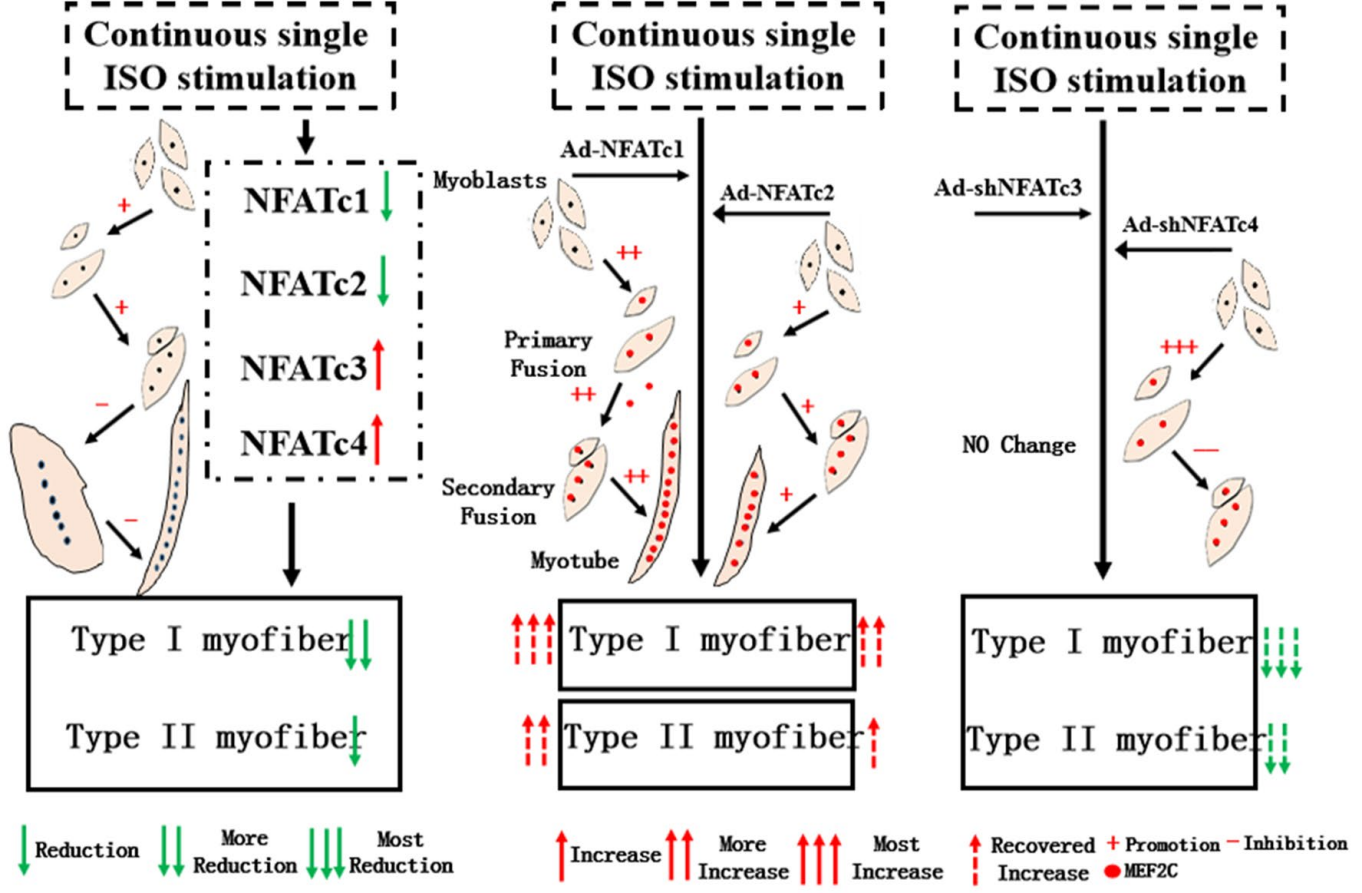
Working model: Continuous exposure to isoprenaline inhibited myoblast differentiation and fusion and altered muscle fiber specialization through the NFAT-MEF2C signaling pathway. NFATc1 signaling controlled the changes in ISO-reduced type I muscle fibers with the help of MEF2C; NFATc4 signaling controlled the changes in type II muscle fibers mediated by ISO in an MEF2C-independent manner.

Due to long-term overactivity of the sympathetic nerve, injury induced by increased levels of NE and E often ascribes to β-AdR1 activation^7^. Typically, isoproterenol (ISO) has been used to imitate the role of β-AdR. This is because of its more stable binding property to β-AdR compared to that of NE and E to both α-AdR and β-AdR, which results in difficulty in analyzing AdR subtype functions^2^. In earlier studies, a single high dose of ISO (10^−4^ M) was usually used to trigger skeletal muscle atrophy^25–27^. Herein, we first found that continuous single-dose ISO at a concentration below 10^−4^ M evidently hindered C2C12 myoblast differentiation and fusion compared with single-dose or interval single-dose administration (Fig. 1A–B). Second, ISO administration with a continuous single dose reduced the nuclear levels of NFATc1/c2 and MyoG/MEF2C and increased the nuclear NFATc3/c4 levels. Finally, continuous single-dose ISO impeded C2C12 myoblast differentiation and fusion, causing a reduction in myotube types I/II by restraining the NFAT-MyoG/MEF2C signaling pathway (Fig. 10).

Continuous single-dose ISO has shown typical dose-dependent traits. However, at 10^−8^ M and 10^−5^ M ISO, myoblast differentiation/fusion and signaling molecules displayed obvious differences. For example, 10^−8^ M ISO markedly reduced NFATc1/c2 levels (50–60%) in myoblast cells, and myoblast differentiation/fusion was slightly decreased at 10^−8^ M ISO; however, these differences were not significant compared with the normal control group. Our previous studies have shown that compared with 10^−5^ M ISO, 10^−8^ M ISO did not alter pAKT, p38MAPK, or pERK1/2 levels, which play important roles in myoblast differentiation/fusion^9^. The increased pERK1/2 levels at 10^−5^ M ISO inhibited myoblast differentiation and fusion through the inactivated AKT and activated FOXO1 signaling pathway. These specific effects could be obviously abolished by the ERK1/2 blocker PD98059, in line with Marino’s report^28^, indicating that pERK1/2 levels at 10^−8^ M ISO contributed to partial preservation of myoblast differentiation and fusion. Of interest, at 10^−5^ M ISO, pERK1/2 is still at a high level, while nuclear NFATc1/c2 proteins are at a minimum level, leading to the remarkable inhibition of myoblast differentiation and fusion, consistent with the effect of increased nuclear FOXO1 and comparable inhibition of NFATs on pancreatic β cell dysfunction^29^.

Indeed, four NFATs are involved in myoblast cell pool homeostasis^30,31^, myoblast recruitment^19^, myoblast differentiation^30–36^, myoblast fusion^19,32,36^, and muscle fiber specialization^37–43^. Herein, with ISO inhibiting myoblast differentiation/fusion (Fig. 1A,B), ISO reduced the levels in NFATc1/c2 and comprehensively increased the levels of NFATc3/c4 (Fig. 3A–E), which was consistent with the result that ISO could not completely inhibit the differentiation and fusion of myoblasts (Fig. 2A–C) because it was involved in the increase in NFATc3/c4 levels^35,44–47^. More importantly, for the first time, once the levels of NFATc3 were time-dependently significantly increased following ISO stimulation, the levels of NFATc1 were rapidly reduced (Fig. 4A–E), and the specific effect could be obviously abolished by knockdown of NFATc3 by shRNA. Similarly, Ad-shNFATc3 also increased NFATc2 levels. However, knockdown of NFATc4 by shRNA decreased the levels of NFATc2 while increasing the levels of NFATc1 (Fig. 5C–F). These results could further explain why knockdown of NFATc3 did not change the inhibitory effect of ISO on myoblast differentiation/fusion, while knockdown of NFATc4 worsened myoblast fusion.

Published data have shown that NFATc2 is an important player in the primary control of myoblast recruitment and myoblast fusion^19,30,32^. In addition to regulating the specialization of muscle fibers, NFATc1 could regulate myoblast fusion by promoting NFATc2 expression^48^, in line with our results that Ad-NFATc1 alone substantially increased the levels of NFATc2 (Fig. 5C–F), suggesting that the recovery of myoblast differentiation and fusion by overexpressing NFATc1 could be involved in the partial restoration of NFATc2 in ISO-treated myoblast cells. Of interest, overexpression of NFATc1 reversed the inhibitory effects of ISO on myoblast differentiation and fusion, resulting in increased myotube size, more than NFATc2 overexpression. This difference could be related to the fact that NFATc1 overexpression markedly increased the cytoplasmic and nuclear levels of NFATc2 accompanied by an obvious increase in the nuclear levels of NFATc1. Relatively, NFATc2 overexpression slightly increased the nuclear levels of NFATc1 with a significant increase in NFATc2 levels in the nucleus. Then, unlike the overexpression of NFATc1, NFATc2 overexpression apparently increased the cytoplasmic levels of NFATc3/4. Indeed, NFATc3 promoted myoblast differentiation and fusion, while NFATc4 inhibited it^35,44–47^. We speculated that this high-level accumulation of cytoplasmic NFATc3/4 could be involved in the partial recovery effects of overexpressing NFATc2 on the length and area of myotubes. Although the detailed mechanism needs to be further clarified in the future, ISO inhibited myoblast differentiation/fusion, resulting in diminished myotube size, which was associated with the destroyed coordination of different members of the NFAT family.

Regarding muscle fiber type specification, four NFATs are involved in the control of type I myofibers and slow muscle specialization^37–43^, especially NFATc1^37,38^. NFATc1-mediated slow muscle specialization and the fast to slow myofiber-type switch require MEF2C and MyoD coordination^40–43^, respectively. In this study, continuous single-dose ISO decreased the MEF2C levels within the nucleus, and the specific effects could be eliminated by overexpressing NFATc1 more than overexpressing NFATc2, resulting in stronger recovery of the inhibited type I muscle fiber, indicating that NFATc1 signaling controlled the changes in ISO-reduced type I muscle fiber, at least partly related to the aid of MEF2C. Of interest, in contrast to the results that the activated NFATc3 contributed to slow muscle marker gene Myh7 expression in the coordination of MyoD, knockdown of NFATc3 by shRNA partially recovered the expression of Myh7 in ISO-stimulated myoblasts cells, attributed to the compensatory changes caused by NFATc3 knockdown; that is, knockdown NFATc3 slightly increased the MEF2C levels of myotubes with more than 3 nuclei while raising the nuclear levels of NFATc1/c2. Meanwhile, knockdown NFATc4 decreased the expression of both Myh7 and Myh1/2/4, which was partially consistent with the result that NFATc4 mainly contributed to fast muscle fiber formation characterized by Myh1/2/4^30,38^. The other reasons for these changes could be related to the evidence that knockdown NFATc4 increased the nuclear NFATc1 levels while reducing the nuclear NFATc2/c3 levels, in addition to the fact that the MEF2C levels of myotubes with more than 3 nuclei were not changed. Therefore, ISO reduced type I and II myofibers, which could be associated with the destruction of NFAT synergy.

## Conclusion

Our results provide a novel mechanism by which continuous single-dose ISO dramatically affects myoblast differentiation and fusion, myotube size and muscle fiber specialization through the NFAT-MyoG/MEF2C signaling pathway.

## Supplementary Information

Supplementary Information 1.

Supplementary Information 2.

## Data Availability

Please contact the corresponding author for data requests.

## Abbreviations

AC: Adenylate cyclase
AdR: Adrenergic receptor
DAPI: 4´,6-Diamidino-2-phenylindole
DCM: Dilated cardiomyopathy
DM: Differentiation medium
DMEM: Dulbecco’s modified Eagle’s medium
E: Epinephrine
FBS: Fetal bovine serum
HF: Heart failure
HPF: High-power field
HRP: Horseradish peroxidase
HS: Horse serum
IF: Immunofluorescence
ISO: Isoprenaline
MD: Muscular dystrophy
MyHC: Myosin heavy chain
MEF2C: Myocyte-specific enhancer factor 2C
MyoD: Myogenic differentiation 1
M: Mol/L
NE: Norepinephrine
Myh1: Myosin heavy chain 1
Myh2: Myosin heavy chain 2
Myh4: Myosin heavy chain 4
Myh7: Myosin heavy chain 7
PBS: Phosphate buffered saline
PKA: Protein kinase A
PKA RIα: PKA regulatory subunit Iα
PVDF: Polyvinylidene fluoride
qPCR: Quantitative polymerase chain reaction
SNS: Sympathetic nerve system
