# Supplementary material for: Continuous exposure to isoprenaline reduced myotube size by delaying myoblast differentiation and fusion through the NFAT-MEF2C signaling pathway
Source: Sci Rep. 2023 Jan 9;13:436. doi: 10.1038/s41598-022-22330-w (PMC9829891; doi:10.1038/s41598-022-22330-w)
Supplement: Supplementary file 1 — Supplementary Information 1. [file 41598_2022_22330_MOESM1_ESM.pdf]

Supplemental Figure source results for “Continuous exposure to isoprenaline reduced myotube size by delaying myoblast differentiation and fusion through the NFAT-MEF2C signaling pathway”

Figure 3. Continuous ISO stimulation dosage-dependently altered NFATc1 and NFATc2 signaling

(A)Continuous ISO stimulation dosage-dependently reduced nuclear levels of NFATc1 and NFATc2 while slightly increased nuclear NFATc4 levels as determined by western blot, five days after myoblast differentiation following the stimulation of ISO delivered with continuous single-dose. (B-E) Semi-quantitative assay from figure 3A. n=3, $^{*}P<0.05$ vs. Ctrl;;$^{\#}P<0.05$ vs. 10^{-8} M ISO; $^{\$}P<0.05$ vs. 10^{-8} M ISO or 10^{-7} M ISO. $^{\&}P<0.05$ vs. 10^{-8} M ISO, 10^{-7} M ISO or 10^{-5} M ISO.

Figure 3A for NFATc1: the result of three replicates.

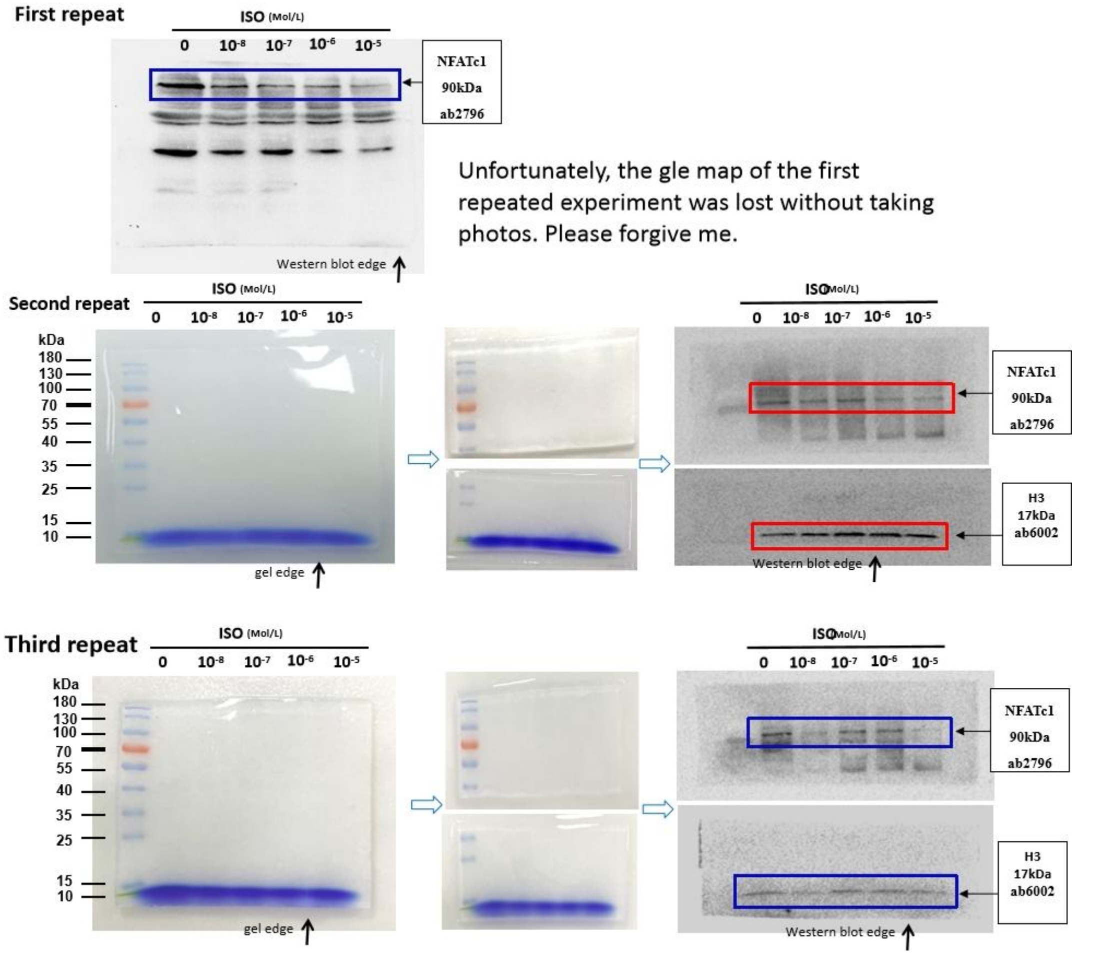

To generate this image, Lysates of myoblasts were analyzed at 6 days of differentiation. First, samples (20 μg proteins) were run on an 10 % SDS-PAGE gel then transferred onto a PVDF membrane (Millipore). Membranes were blocked in 5% nonfat milk in TBS-0.1 % Tween® 20 (TBS-T) before incubation with NFATc1-mouse anti-mouse/human (1:500, ab2796,ABCAM,USA) or H3 mouse-anti mouse /human (1:500, Ab6002, ABCAM,USA) antibody overnight at 4 ℃, respectively. Blots were washed four times in TBS-T, incubated with secondary antibodies for 90

min at room temperature, washed again four times then imaged. Secondary antibodies used were goat anti-mouse IgG H&L(ANT019, antgene.CN) preabsorbed at 1/2000 dilution.
In order to save samples and workload, we divide the glue and film into two, as shown in the figure. Predicted band size: 90 kDa. Observed band size: 75,80,90 kDa.
https://www.abcam.cn/nfat2-antibody-7a6-ab2796.html
https://www.abcam.cn/histone-h3-tri-methyl-k27-antibody-mabcam-6002-chip-grade-ab6002.html

Figure 3A for NFATc2: the result of three replicates.

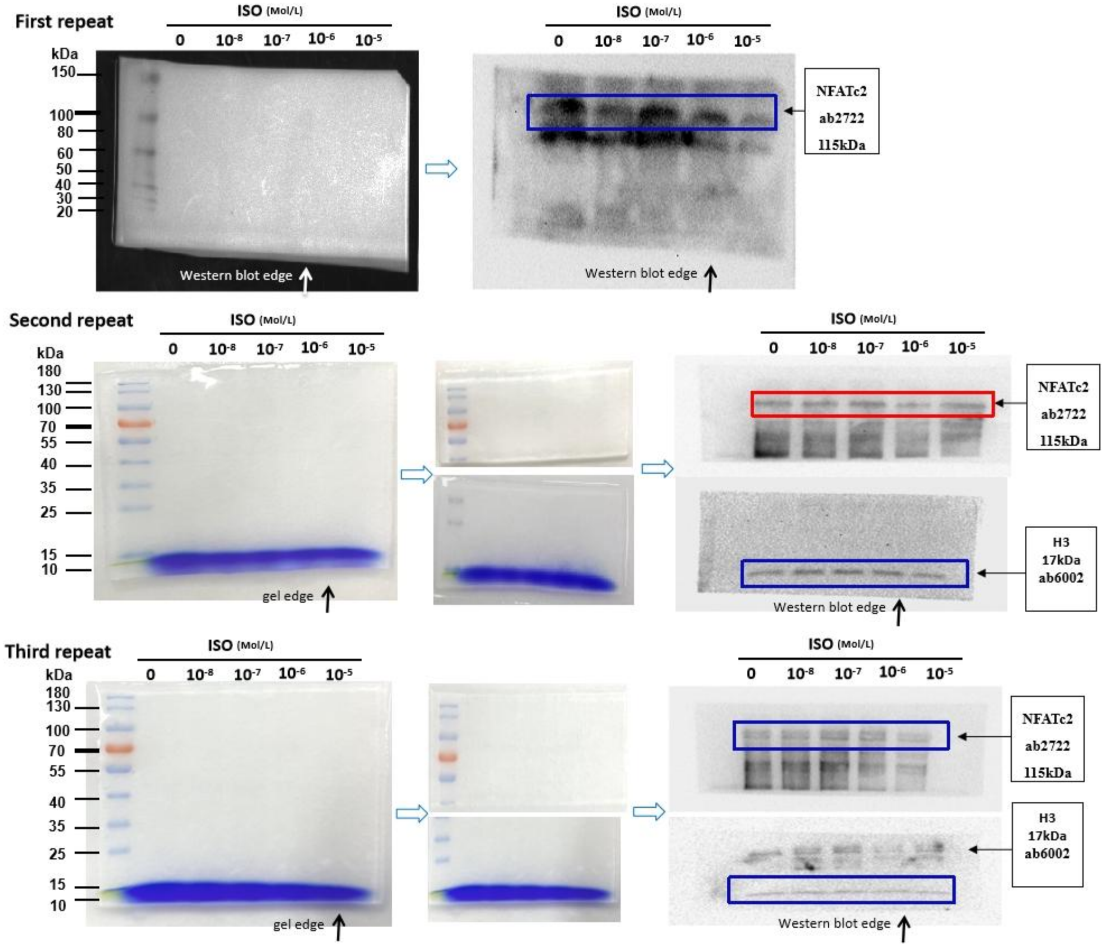

To generate this image, Lysates of myoblasts were analyzed at 6 days of differentiation. First, samples (20 μg proteins) were run on an 10 % SDS-PAGE gel then transferred onto a PVDF membrane (Millipore). Membranes were blocked in 5% nonfat milk in TBS-0.1 % Tween® 20 (TBS-T) before incubation with NFATc2 mouse anti-mouse/human (1:500, ab2722,ABCAM,USA) or H3 mouse-anti mouse /human (1:500, Ab6002, ABCAM,USA) antibody overnight at 4 ℃, respectively. Blots were washed four times in TBS-T, incubated with secondary antibodies for 90 min at room temperature, washed again four times then imaged. Secondary antibodies used were goat anti-mouse IgG H&L(ANT019, antgene.CN) preabsorbed at 1/2000 dilution.
In order to save samples and workload, we divide the glue and film into two, as shown in the figure.
Predicted band size for NFATc2(NFAT1): 115 kDa; Observed band size: 150 kDa. Additional bands at 62 kDa. We are unsure as to the identity of these extra bands.
Observed band size for H3: 17 kDa.

https://www.abcam.cn/nfat1-antibody-25a10d6d2-ab2722.html
https://www.abcam.cn/histone-h3-tri-methyl-k27-antibody-mabcam-6002-chip-grade-ab6002.html
NFATc3: the result of three replicates

Figure 3A for NFATc3: the result of three replicates.

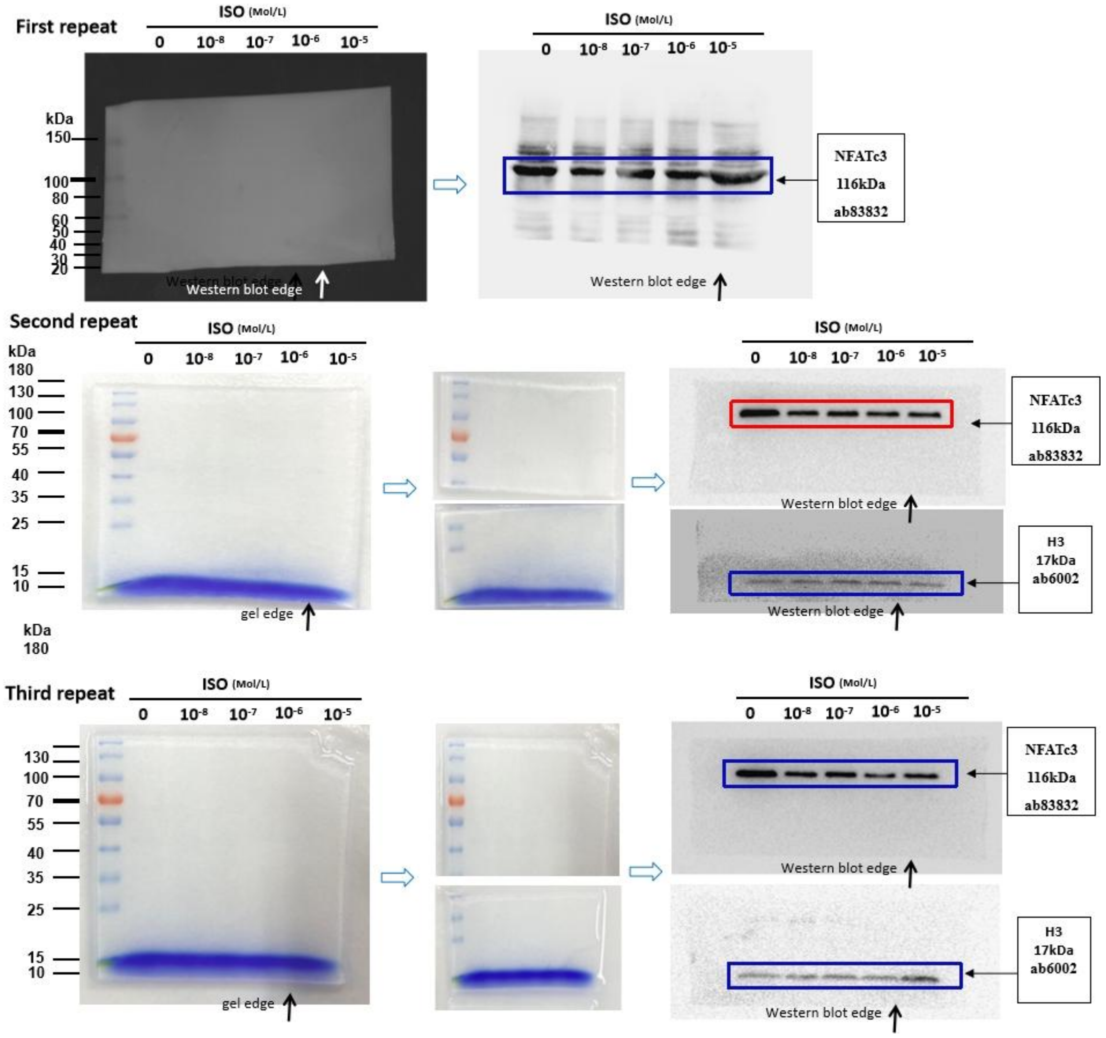

To generate this image, Lysates of myoblasts were analyzed at 6 days of differentiation. First, samples (20 μg proteins) were run on an 10 % SDS-PAGE gel then transferred onto a PVDF membrane (Millipore). Membranes were blocked in 5% nonfat milk in TBS-0.1 % Tween® 20 (TBS-T) before incubation with NFATc3 rabbit anti-mouse/human (1:500, ab83832,ABCAM,USA) or H3 mouse-anti mouse /human (1:500, Ab6002, ABCAM,USA) antibody overnight at 4 ℃, respectively. Blots were washed four times in TBS-T, incubated with secondary antibodies for 90 min at room temperature, washed again four times then imaged. Secondary antibodies used were goat anti-rabbit IgG H&L (ANT020, antgene.CN) preabsorbed and goat anti-mouse IgG H&L(ANT019, antgene.CN) preabsorbed at 1/2000 dilution.

In order to save samples and workload, we divide the glue and film into two, as shown in the figure.

Observed band size for NFATc3: 116 kDa. Observed band size for H3: 17 kDa.

https://www.abcam.cn/nfat4nf-atc3-antibody-ab83832.html
https://www.abcam.cn/histone-h3-tri-methyl-k27-antibody-mabcam-6002-chip-grade-ab6002.html

Figure 3A for NFATc4: the result of three replicates.

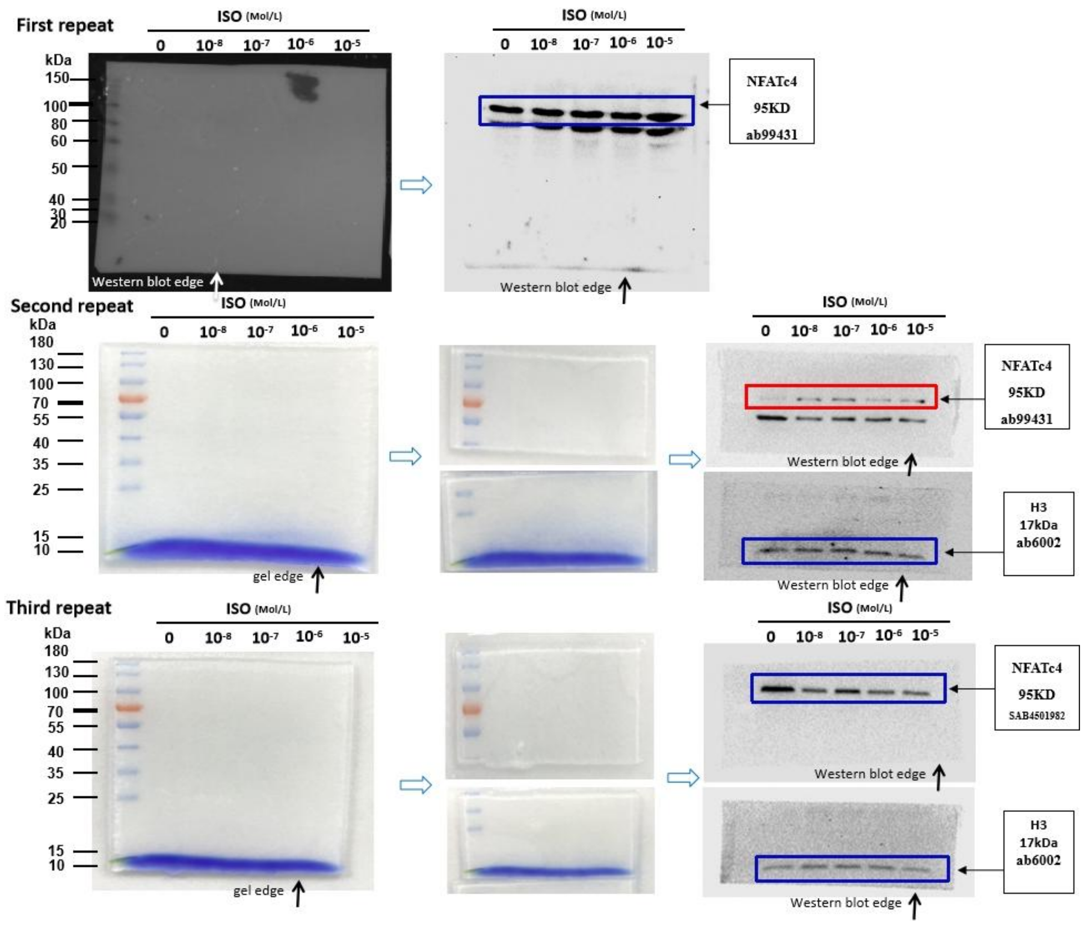

To generate this image, Lysates of myoblasts were analyzed at 6 days of differentiation. First, samples (20 μg proteins) were run on an 10 % SDS-PAGE gel then transferred onto a PVDF membrane (Millipore). Membranes were blocked in 5% nonfat milk in TBS-0.1 % Tween® 20 (TBS-T) before incubation with NFATc4 rabbit anti-mouse/human (1:1000,ab99431, ABCAM, USA; 1:1000, SAB4501982, SIGMA, USA) or H3 mouse-anti mouse /human (1:500, Ab6002, ABCAM,USA) antibody overnight at 4 ℃, respectively. Blots were washed four times in TBS-T, incubated with secondary antibodies for 90 min at room temperature, washed again four times then imaged. Secondary antibodies used were goat anti-rabbit IgG H&L (ANT020, antgene.CN) preabsorbed and goat anti-mouse IgG H&L (ANT019, antgene.CN) preabsorbed at 1/10000 dilution.

In order to save samples and workload, we divide the glue and film into two, as shown in the figure.

Observed band size for NFATc4: 95 kDa. Additional bands at 71 kDa using ab99431. We are unsure as to the identity of these extra bands. And then, we used SAB4501982 to further confirm it that observed band size for NFATc4 was 95 kDa. Observed band size for H3: 17 kDa.

https://www.abcam.cn/nfatc4-antibody-ab99431.html

https://www.sigmaaldrich.cn/CN/zh/product/sigma/sab4501982

https://www.abcam.cn/histone-h3-tri-methyl-k27-antibody-mabcam-6002-chip-grade-ab6002.html

Figure 3A for H3: the result of three replicates. Please check them in above results.

Figure 4. Continuous ISO stimulation time-dependently altered NFATc1 and NFATc2 signaling

(A) Continuous ISO stimulation reduced nuclear levels of NFATc1 and NFATc2 while slightly increased nuclear levels of NFATc3 and NFATc4 as determined by western blot, 2, 4 and 6 days after myoblast differentiation following the stimulation of ISO delivered with continuous single dose. (B-E) Semi-quantitative assay from figure 3A. Three independently repeated experiments were performed. n=3, $^{*}P < 0.05$ vs. Ctrl at indicated time; $^{\&}P < 0.05$ vs. 10^{-5} M ISO group on the second day of myoblast differentiation; $^{\#}P < 0.05$ vs. 10^{-5} M ISO group on the 4th day of myoblast differentiation.

Figure 4A for NFATc1: the result of three replicates.

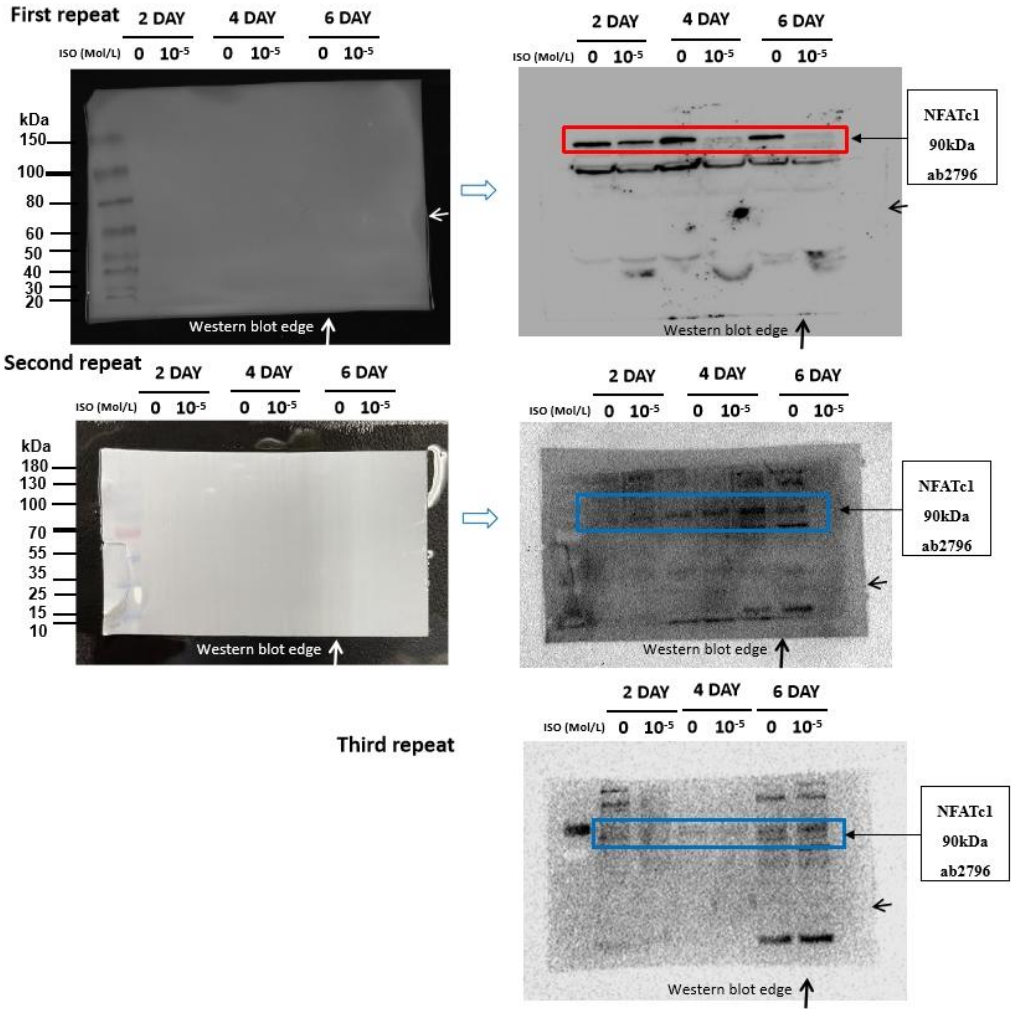

To generate this image, Lysates of myoblasts were analyzed at 6 days of differentiation. First, samples (20 µg proteins) were run on an 10 % SDS-PAGE gel then transferred onto a PVDF membrane (Millipore). Membranes were blocked in 5% nonfat milk in TBS-0.1 % Tween® 20 (TBS-T) before incubation with NFATc1-mouse anti-mouse/human (1:500, ab2796,ABCAM,USA) antibody overnight at 4 ℃, respectively. Blots were washed four times in TBS-T, incubated with secondary antibodies for 90 min at room temperature, washed again four times then imaged.

Secondary antibodies used were Goat anti-Mouse IgG H&L preabsorbed (ANT019, antgene, CN) preabsorbed at 1/2000 dilution.
Predicted band size: 90 kDa. Observed band size: 75,80,90 kDa.
https://www.abcam.cn/nfat2-antibody-7a6-ab2796.html
https://www.abcam.cn/histone-h3-tri-methyl-k27-antibody-mabcam-6002-chip-grade-ab6002.html

Figure 4A for NFATc2: the result of three replicates.

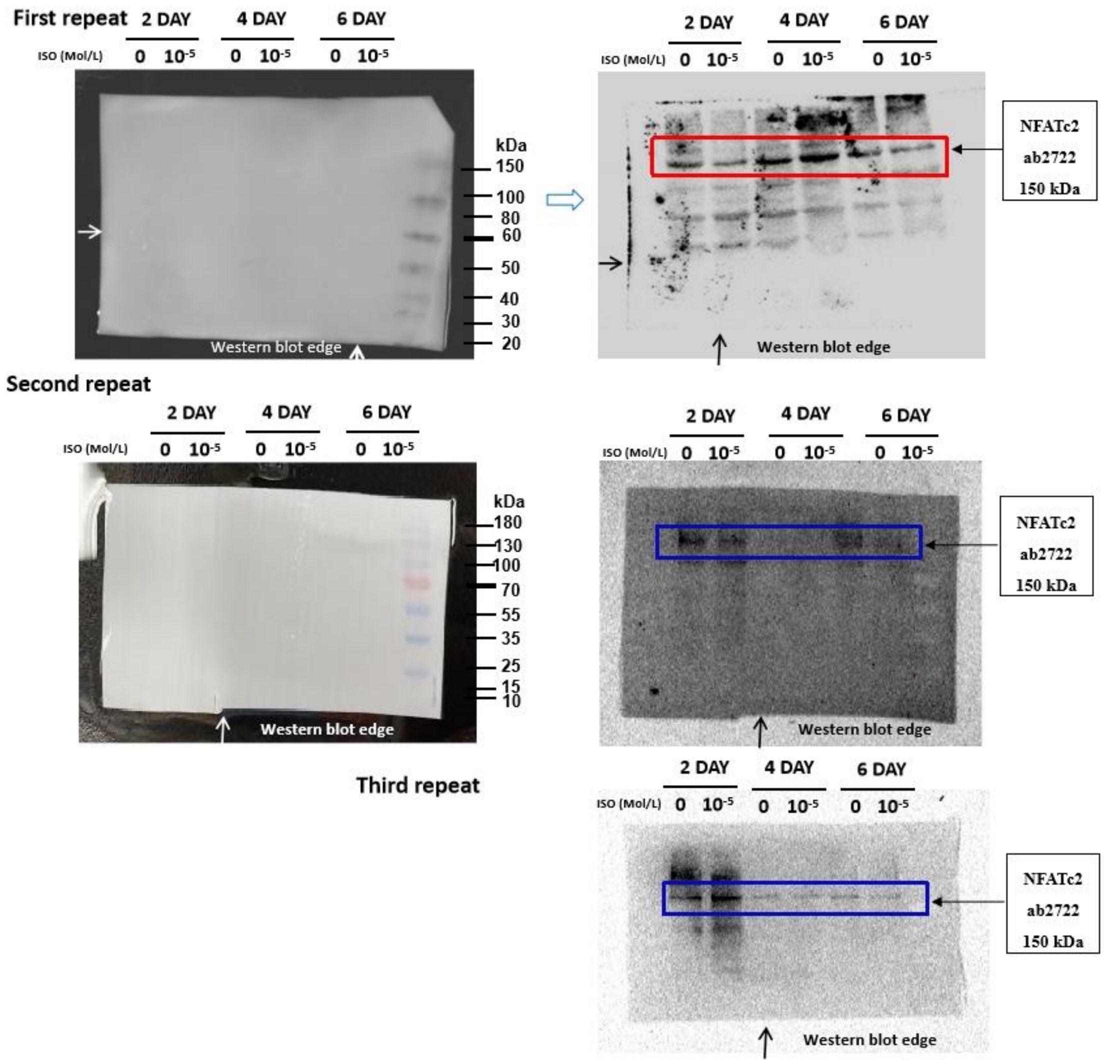

To generate this image, Lysates of myoblasts were analyzed at 6 days of differentiation. First, samples (20 µg proteins) were run on an 10 % SDS-PAGE gel then transferred onto a PVDF membrane (Millipore). Membranes were blocked in 5% nonfat milk in TBS-0.1 % Tween® 20 (TBS-T) before incubation with NFATc2-mouse anti-mouse/human (1:500, ab2722,ABCAM,USA) antibody overnight at 4 ℃, respectively. Blots were washed four times in TBS-T, incubated with secondary antibodies for 90 min at room temperature, washed again four times then imaged. Secondary antibodies used were goat anti-mouse IgG H&L(ANT019, antgene.CN) preabsorbed at 1/2000 dilution.
Predicted band size for NFATc2(NFAT1): 115 kDa; Observed band size: 150 kDa. Additional bands at 62 kDa. We are unsure as to the identity of these extra bands.

https://www.abcam.cn/nfat1-antibody-25a10d6d2-ab2722.html
https://www.abcam.cn/histone-h3-tri-methyl-k27-antibody-mabcam-6002-chip-grade-ab6002.html

Figure 4A for NFATc3: the result of three replicates.

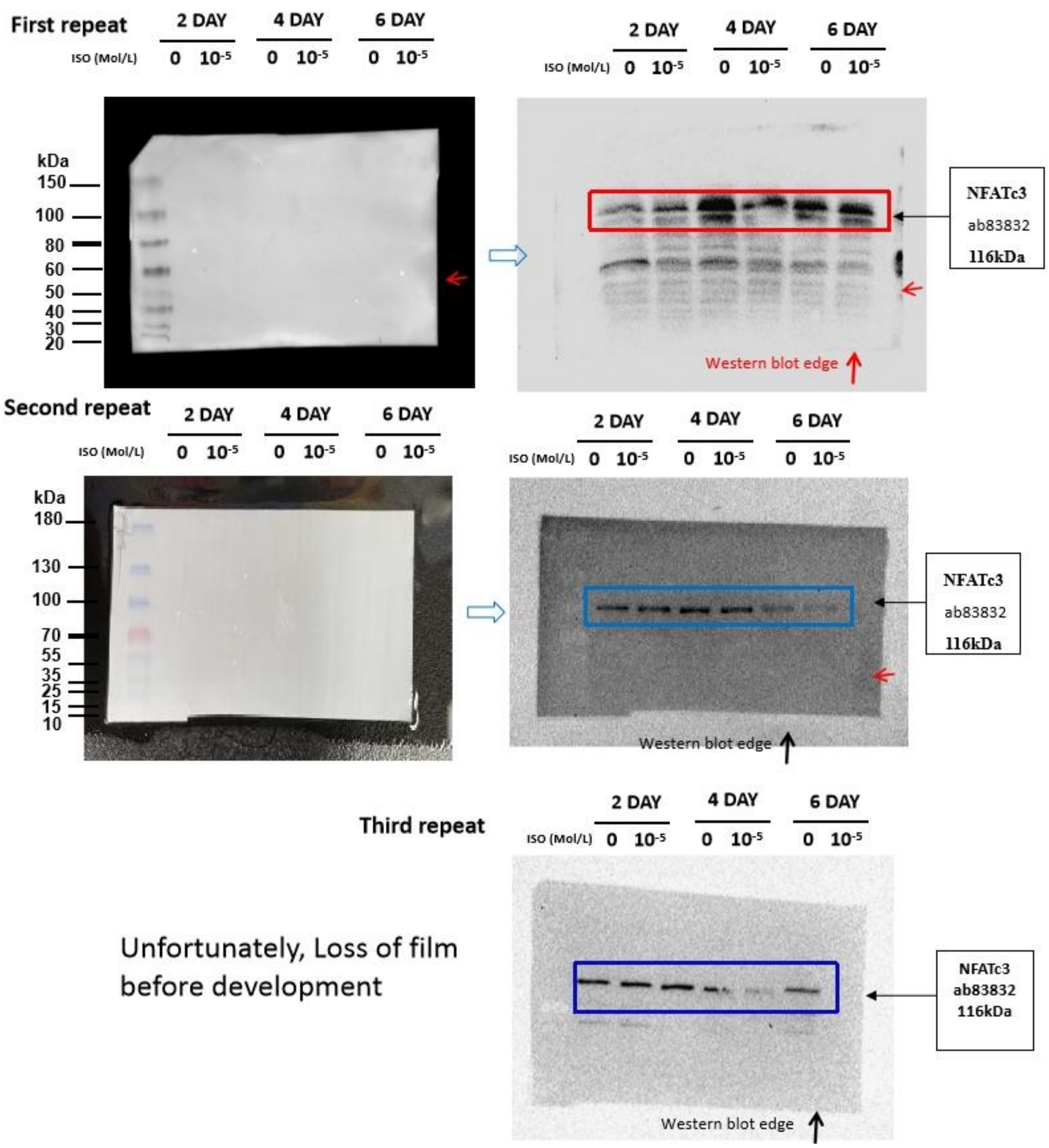

To generate this image, Lysates of myoblasts were analyzed at 6 days of differentiation. First, samples (20 μg proteins) were run on an 10 % SDS-PAGE gel then transferred onto a PVDF membrane (Millipore). Membranes were blocked in 5% nonfat milk in TBS-0.1 % Tween® 20 (TBS-T) before incubation with NFATc3 rabbit anti-mouse/human (1:500, ab83832,ABCAM,USA) antibody overnight at 4 ℃, respectively. Blots were washed four times in TBS-T, incubated with secondary antibodies for 90 min at room temperature, washed again four times then imaged. Secondary antibodies used were goat anti-rabbit IgG H&L (ANT020, antgene.CN) preabsorbed at 1/2000 dilution. Observed band size for NFATc3: 116 kDa. Observed band size for H3: 17 kDa.
https://www.abcam.cn/nfat4nf-atc3-antibody-ab83832.html

https://www.abcam.cn/histone-h3-tri-methyl-k27-antibody-mabcam-6002-chip-grade-ab6002.html

Figure 4A for NFATc4: the result of three replicates.

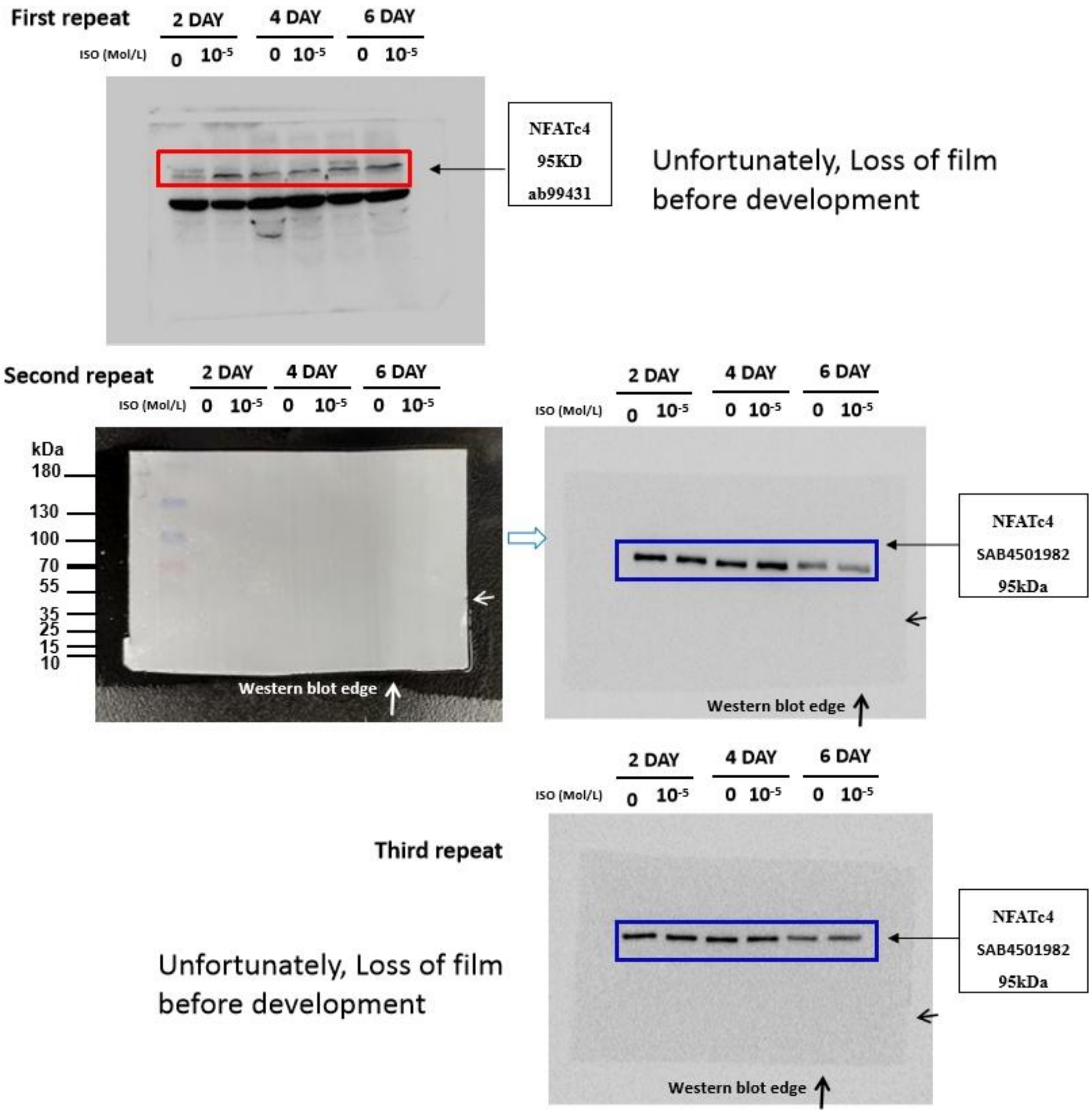

To generate this image, Lysates of myoblasts were analyzed at 6 days of differentiation. First, samples (20 μg proteins) were run on an 10 % SDS-PAGE gel then transferred onto a PVDF membrane (Millipore). Membranes were blocked in 5% nonfat milk in TBS-0.1 % Tween® 20 (TBS-T) before incubation with NFATc4 rabbit anti-mouse/human (1:1000,ab99431, ABCAM, USA; 1:1000, SAB4501982, SIGMA, USA) or H3 mouse-anti mouse /human (1:500, Ab6002, ABCAM,USA) antibody overnight at 4 ℃, respectively. Blots were washed four times in TBS-T, incubated with secondary antibodies for 90 min at room temperature, washed again four times then imaged. Secondary antibodies used were goat anti-rabbit IgG H&L (ANT020, antgene.CN) preabsorbed and goat anti-mouse IgG H&L (ANT019, antgene.CN) preabsorbed at 1/10000 dilution.

Observed band size for NFATc4: 95 kDa. Additional bands at 71 kDa using ab99431. We are unsure as to the identity of these extra bands. And then, we used SAB4501982 to further confirm it that observed band size for NFATc4 was 95 kDa.

https://www.abcam.cn/nfatc4-antibody-ab99431.html
https://www.sigmaaldrich.cn/CN/zh/product/sigma/sab4501982
Figure 4A for H3: the result of three replicates.

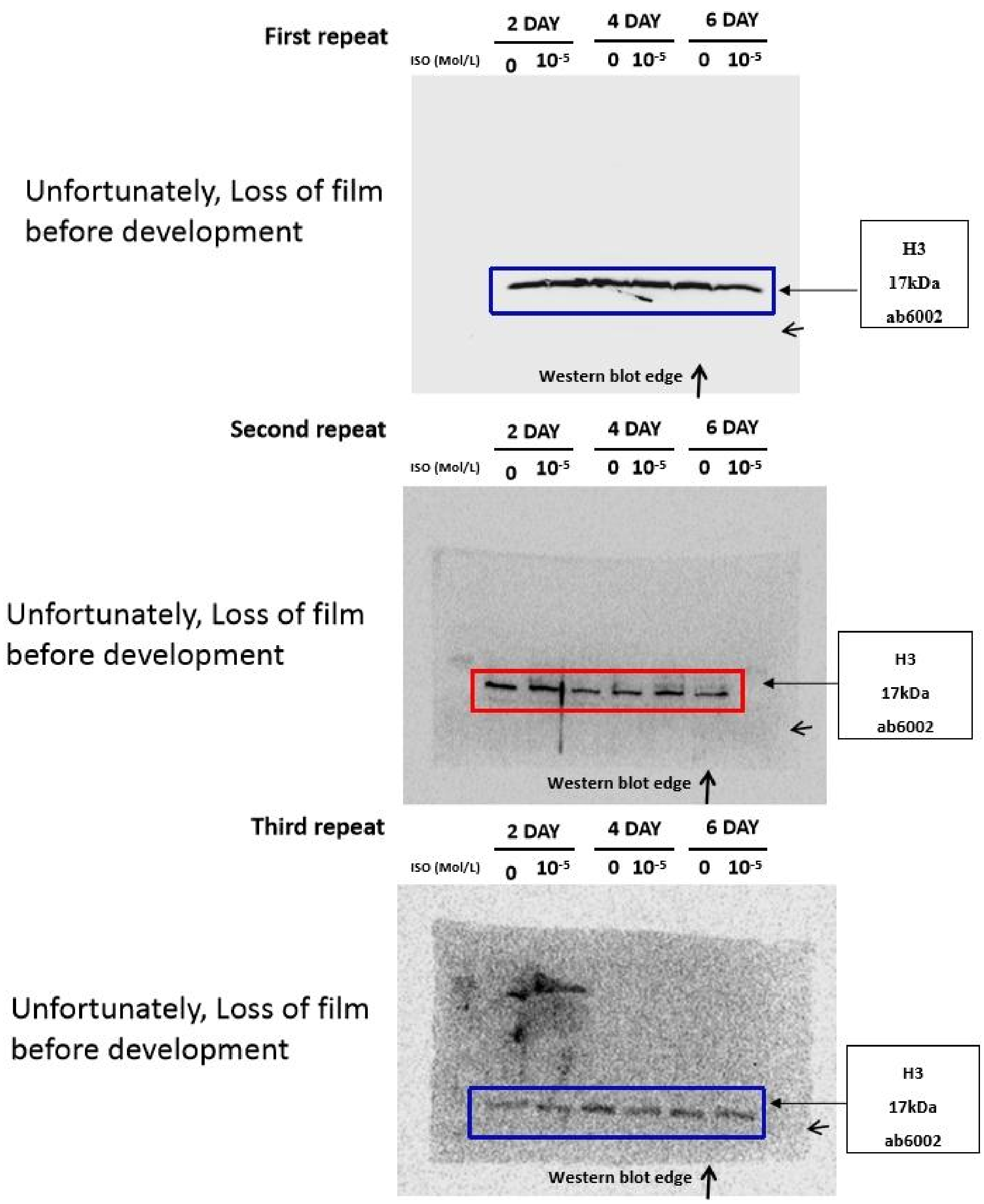

To generate this image, Lysates of myoblasts were analyzed at 6 days of differentiation. First, samples (20 μg proteins) were run on an 10 % SDS-PAGE gel then transferred onto a PVDF membrane (Millipore). Membranes were blocked in 5% nonfat milk in TBS-0.1 % Tween® 20 (TBS-T) before incubation with H3 mouse-anti mouse /human (1:500, Ab6002, ABCAM,USA) antibody overnight at 4 ℃, respectively. Blots were washed four times in TBS-T, incubated with secondary antibodies for 90 min at room temperature, washed again four times then imaged. Secondary antibodies used were goat anti-mouse IgG H&L (ANT019, antgene.CN) preabsorbed at 1/10000 dilution.

Observed band size for H3: 17 kDa.
https://www.abcam.cn/histone-h3-tri-methyl-k27-antibody-mabcam-6002-chip-grade-ab6002.html

Figure 5. Transfection efficiency of over-expression or knockdown of NFAT into C2C12 myoblast cell.

(A)Western blot showed that adenovirus vector was successfully transferred into myoblasts by detecting its Flag-His after it were transfected with 100 optimal multiplications of infection (MOI) for indicated adenovirus expression vectors. (B-E)Quantitative analysis of transfection efficiency of these specific adenovirus into C2C12 myoblast cells were determined by qPCR after myoblast cells were transfected with adenovirus mediated over-expression of NFATc1 and NFATc2, or knockdown of NFATc3 and NFATc4 by shRNA (100 MOI) for 72 h. n=3, $^{*}P < 0.05$ vs. Ad-Null. (F) Cytoplasmic proteins were evaluated by using western blot. (G) Semi-quantitative assay from Figure 5C. Three independently repeated experiments were performed. n=3, $^{*}P < 0.05$ vs. DM;$^{\&}P < 0.05$ vs. 10^{-5} M ISO group; $^{\#}P < 0.05$ vs. 10^{-5} M ISO group; $^{\$}P < 0.05$ vs. 10^{-5} M ISO+Ad-shNFATc3 group. (H-I) Western blot and semi-quantitative assay was used to evaluate nucleus proteins in these adenoviruses treated cells. n=3, $^{*}P < 0.05$ vs. DM;$^{\&}P < 0.05$ vs. 10^{-5} M ISO group; $^{\#}P < 0.05$ vs. 10^{-5} M ISO group; $^{\$}P < 0.05$ vs. 10^{-5} M ISO+Ad-shNFATc3 group;$^{\wedge}P < 0.05$ vs. DM.

Figure 5F for Flag-His: the result of one time.

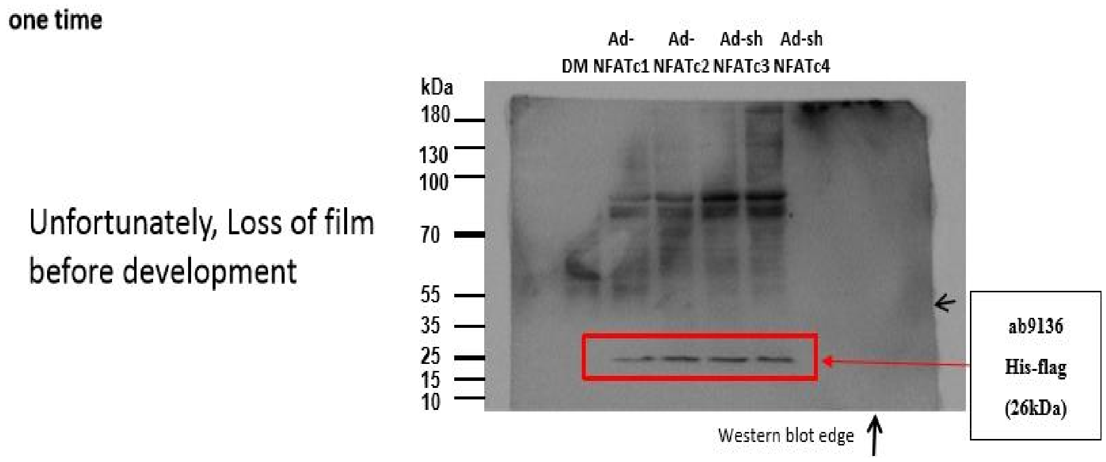

To generate this image, Lysates of myoblasts were analyzed at 6 days of differentiation. First, samples (20 μg proteins) were run on an 10 % SDS-PAGE gel then transferred onto a PVDF membrane (Millipore). Membranes were blocked in 5% nonfat milk in TBS-0.1 % Tween® 20 (TBS-T) before incubation with His tag-goat anti-mouse (1:1000, ab9136,ABCAM,USA) antibody overnight at 4 ℃, respectively. Blots were washed four times in TBS-T, incubated with secondary antibodies for 90 min at room temperature, washed again four times then imaged. Secondary antibodies used were rabbit anti-goat IgG H&L(ANT021, antgene.CN) preabsorbed at 1/2000 dilution.
Observed band size: 26 kDa.

https://www.abcam.cn/6x-his-tag-antibody-ab9136.html

Figure 5F for NFATc1: the result of three replicates.

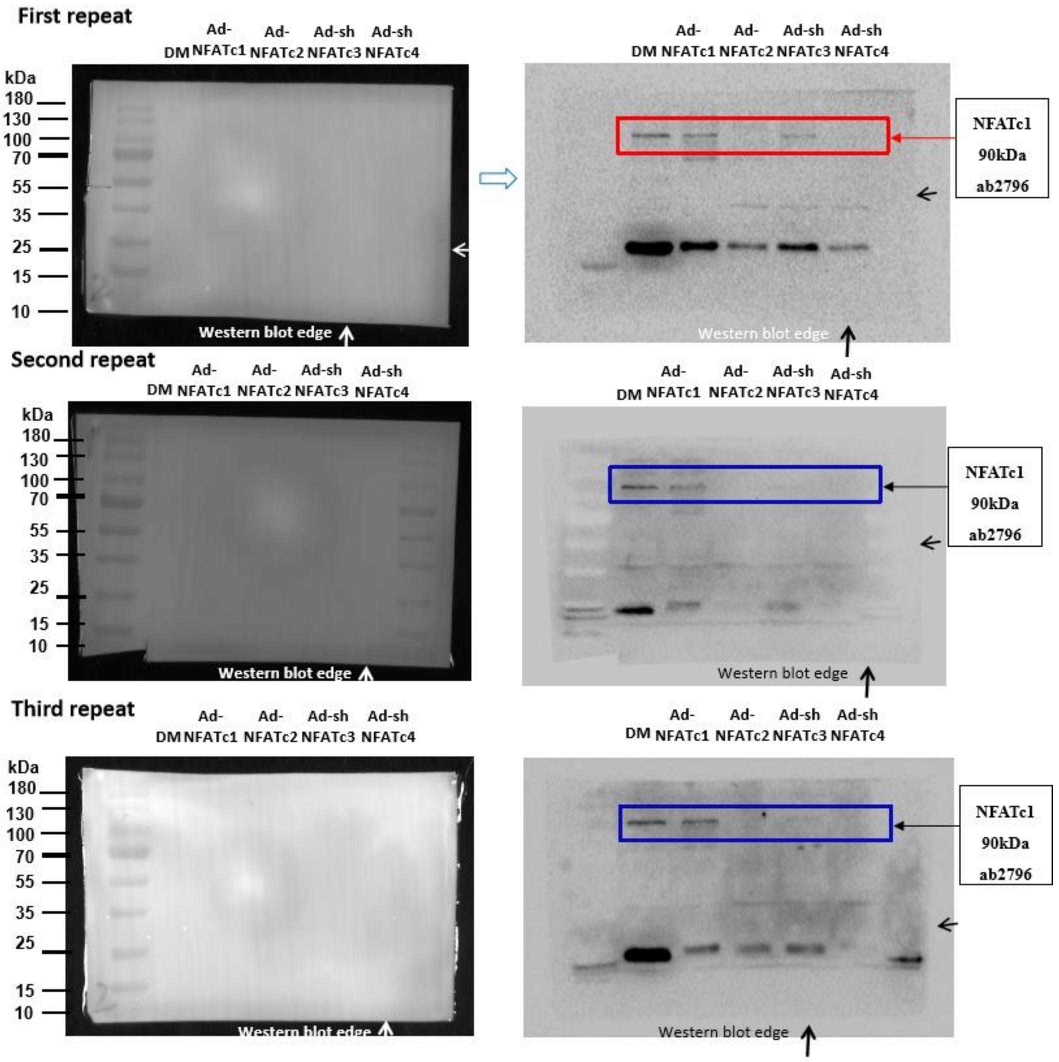

To generate this image, Lysates of myoblasts were analyzed at 6 days of differentiation. First, samples (20 μg proteins) were run on an 10 % SDS-PAGE gel then transferred onto a PVDF membrane (Millipore). Membranes were blocked in 5% nonfat milk in TBS-0.1 % Tween® 20 (TBS-T) before incubation with NFATc1-mouse anti-mouse/human (1:500, ab2796,ABCAM,USA) antibody overnight at 4 ℃, respectively. Blots were washed four times in TBS-T, incubated with secondary antibodies for 90 min at room temperature, washed again four times then imaged. Secondary antibodies used were d goat anti-mouse IgG H&L(ANT019, antgene.CN) preabsorbed at 1/2000 dilution.

Observed band size: 75,80,90 kDa

https://www.abcam.cn/nfat2-antibody-7a6-ab2796.html

Figure 5F for NFATc2: the result of three replicates.

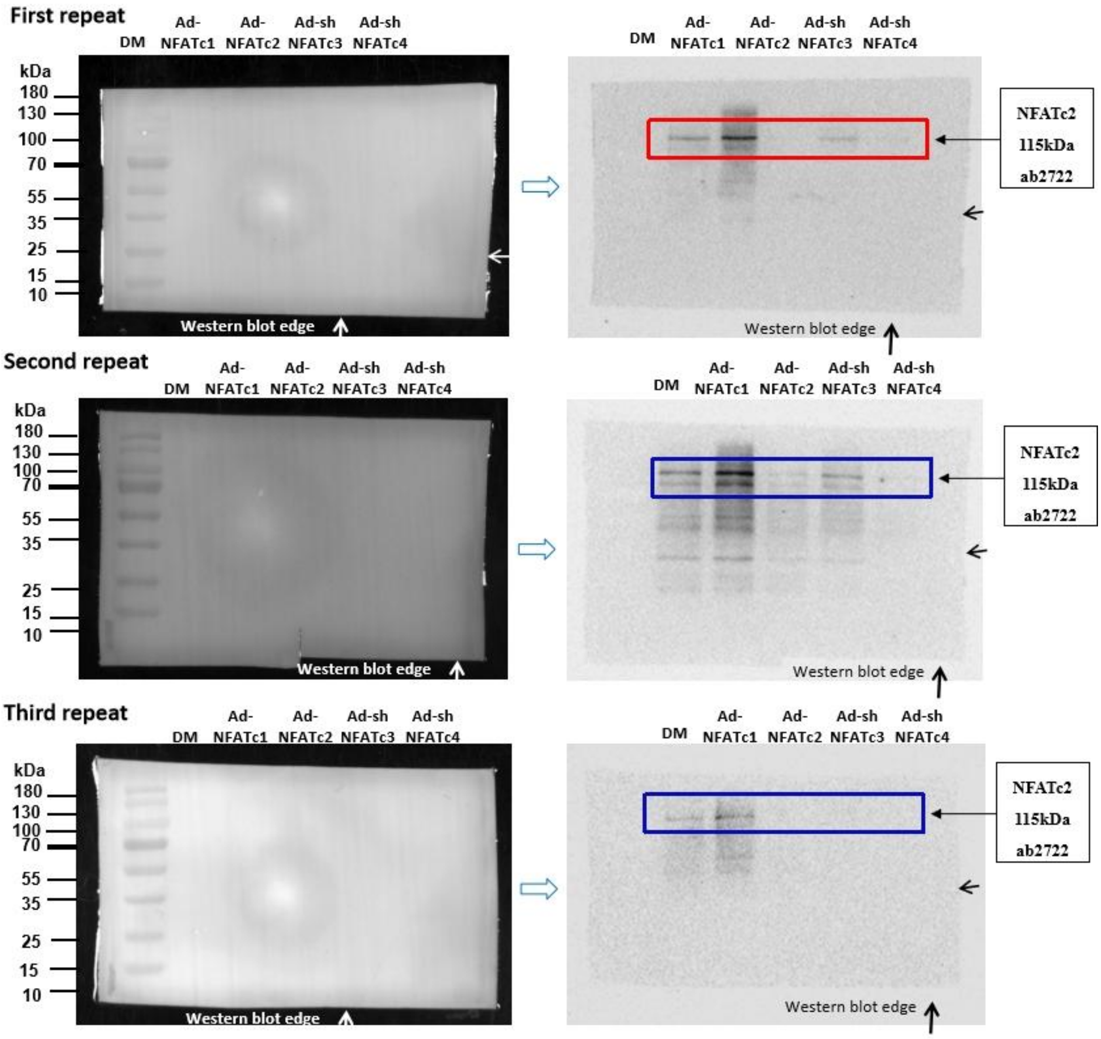

To generate this image, Lysates of myoblasts were analyzed at 6 days of differentiation. First, samples (20 μg proteins) were run on an 10 % SDS-PAGE gel then transferred onto a PVDF membrane (Millipore). Membranes were blocked in 5% nonfat milk in TBS-0.1 % Tween® 20 (TBS-T) before incubation with NFATc2-mouse anti-mouse/human (1:500, ab2722,ABCAM,USA) antibody overnight at 4 ℃, respectively. Blots were washed four times in TBS-T, incubated with secondary antibodies for 90 min at room temperature, washed again four times then imaged. Secondary antibodies used were goat anti-mouse IgG H&L(ANT019, antgene.CN) preabsorbed at 1/10000 dilution.

Predicted band size: 115 kDa; Observed band size: 150 kDa.

https://www.abcam.cn/nfat1-antibody-25a10d6d2-ab2722.html

Figure 5F for NFATc3: the result of three replicates.

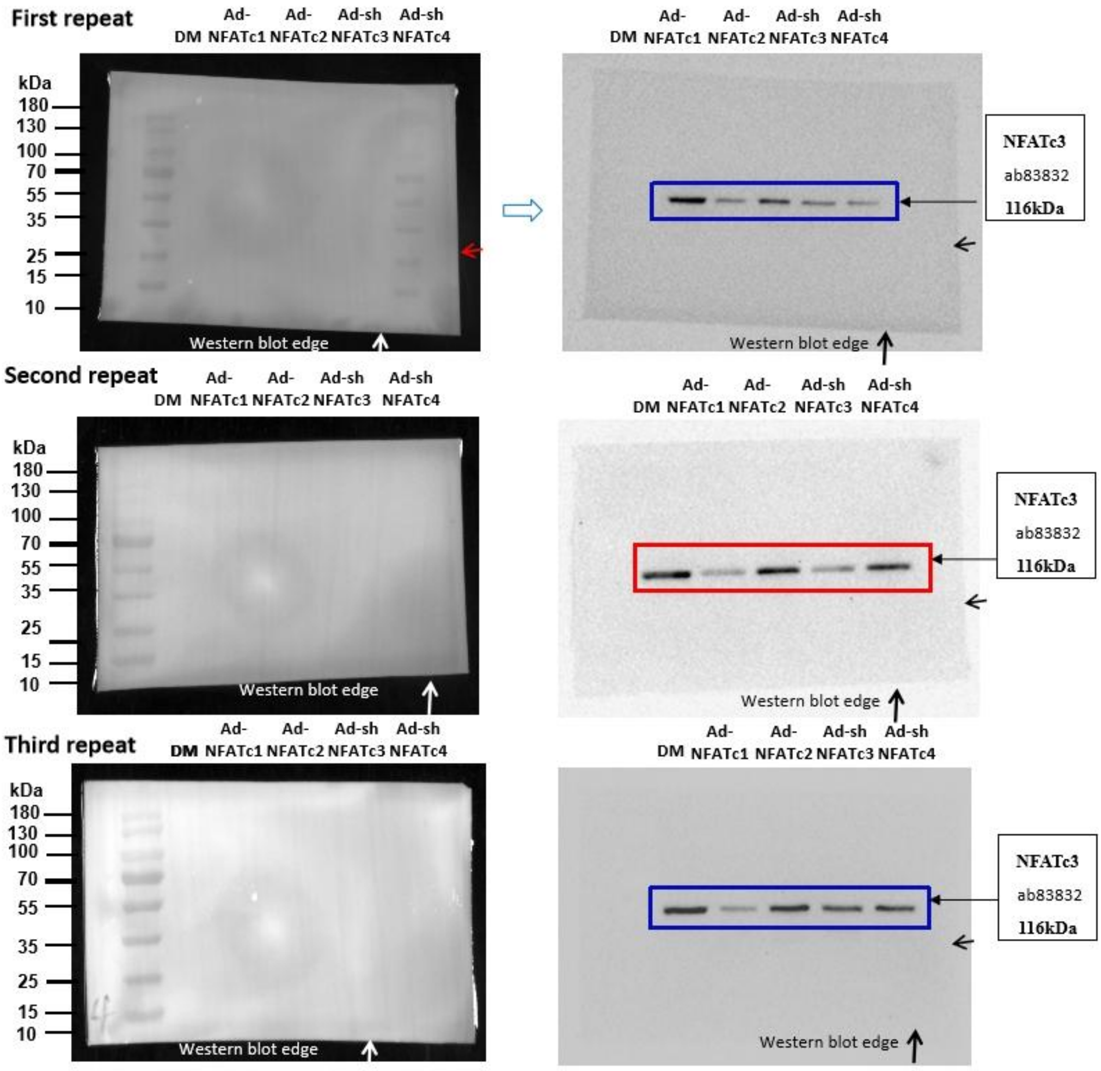

To generate this image, Lysates of myoblasts were analyzed at 6 days of differentiation. First, samples (20 μg proteins) were run on an 10 % SDS-PAGE gel then transferred onto a PVDF membrane (Millipore). Membranes were blocked in 5% nonfat milk in TBS-0.1 % Tween® 20 (TBS-T) before incubation with NFATc3 rabbit anti-mouse/human (1:500, ab83832,ABCAM,USA) antibody overnight at 4 ℃, respectively. Blots were washed four times in TBS-T, incubated with secondary antibodies for 90 min at room temperature, washed again four times then imaged. Secondary antibodies used were goat anti-rabbit IgG H&L (ANT020, antgene.CN) preabsorbed at 1/2000 dilution.

Observed band size for NFATc3: 116 kDa.

https://www.abcam.cn/nfat4nf-atc3-antibody-ab83832.html

Figure 5F for NFATc4: the result of three replicates.

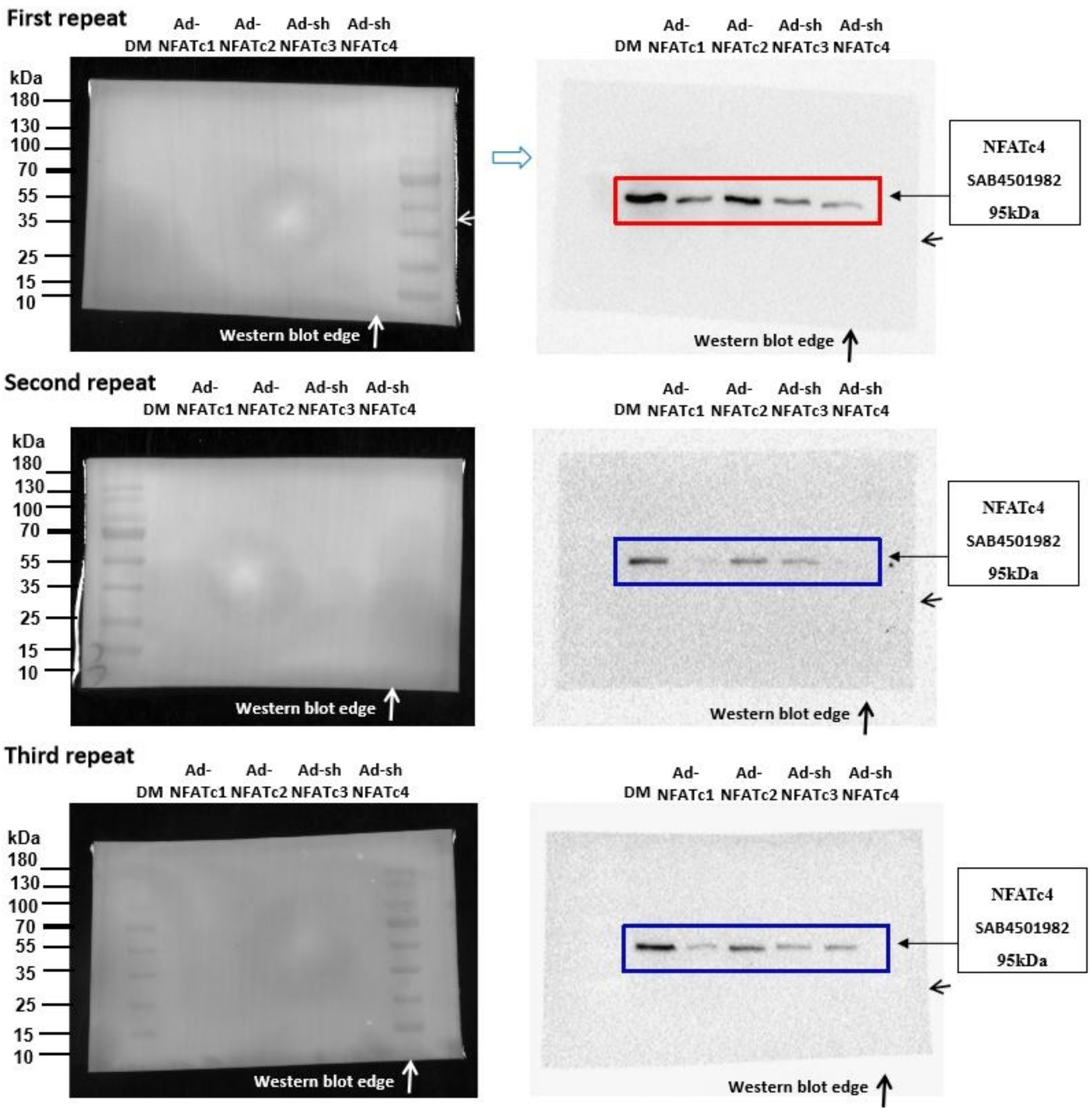

To generate this image, Lysates of myoblasts were analyzed at 6 days of differentiation. First, samples (20 μg proteins) were run on an 10 % SDS-PAGE gel then transferred onto a PVDF membrane (Millipore). Membranes were blocked in 5% nonfat milk in TBS-0.1 % Tween® 20 (TBS-T) before incubation with NFATc4 rabbit anti-mouse/human (1:1000,ab99431, ABCAM, USA; 1:1000, SAB4501982, SIGMA, USA) antibody overnight at 4 ℃, respectively. Blots were washed four times in TBS-T, incubated with secondary antibodies for 90 min at room temperature, washed again four times then imaged. Secondary antibodies used were goat anti-rabbit IgG H&L (ANT020, antgene.CN) preabsorbed at 1/10000 dilution.

In order to save samples and workload, we divide the glue and film into two, as shown in the figure.

Observed band size for NFATc4: 95 kDa.

https://www.sigmaaldrich.cn/CN/zh/product/sigma/sab4501982

Figure 5F for NFATc4: the result of three replicates.

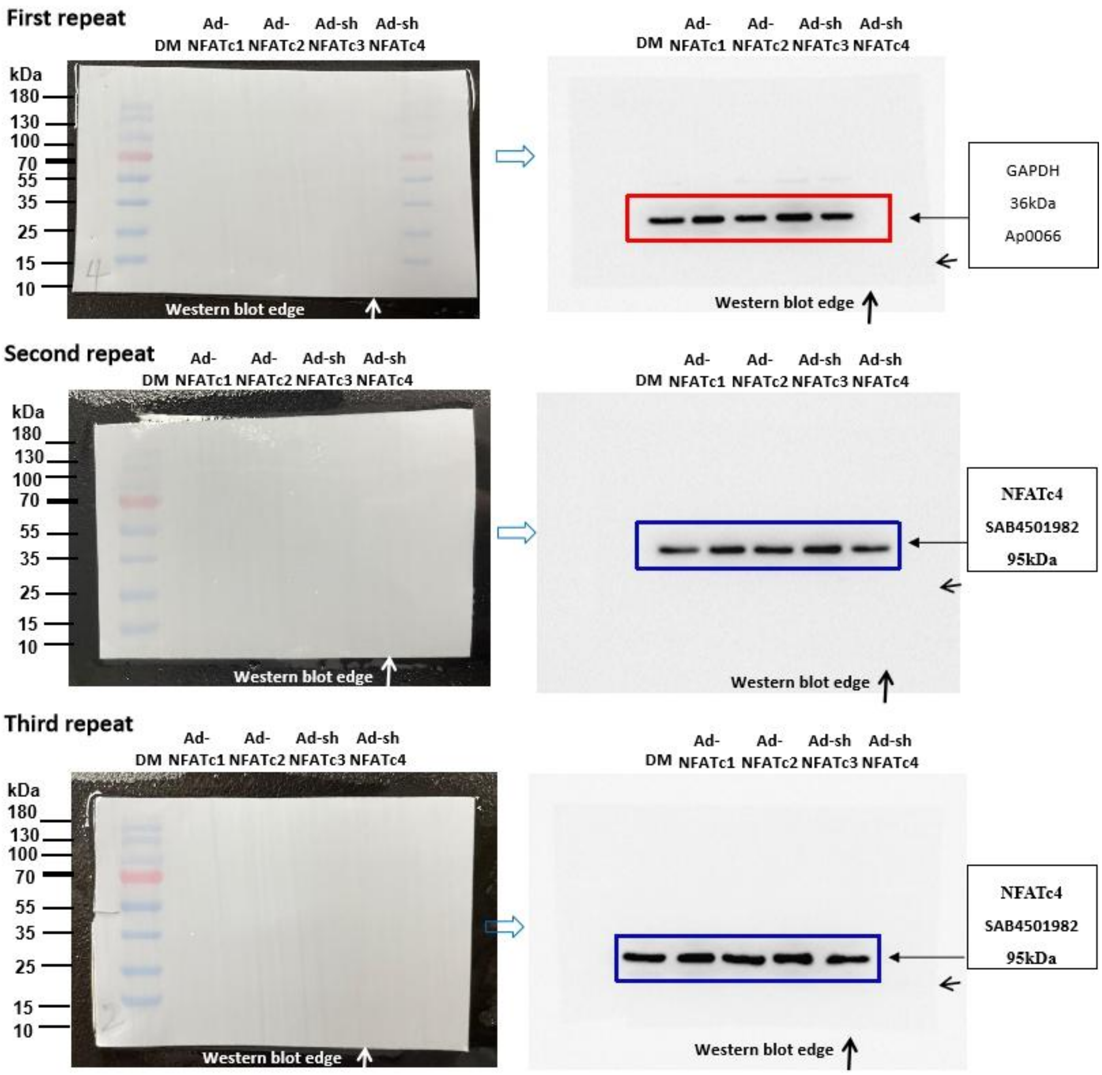

To generate this image, Lysates of myoblasts were analyzed at 6 days of differentiation. First, samples (20 μg proteins) were run on an 10 % SDS-PAGE gel then transferred onto a PVDF membrane (Millipore). Membranes were blocked in 5% nonfat milk in TBS-0.1 % Tween® 20 (TBS-T) before incubation with GAPDH rabbit anti-mouse/human (1:500, Ap0066, Bioworld) antibody overnight at 4 ℃, respectively. Blots were washed four times in TBS-T, incubated with secondary antibodies for 90 min at room temperature, washed again four times then imaged. Secondary antibodies used were goat anti-rabbit IgG H&L (ANT020, antgene.CN) preabsorbed at 1/10000 dilution.

Observed band size for GAPDH: 36 kDa

http://www.bioworlde.com/upimages/AP0066.

Figure 5H for NFATc1: the result of three replicates.

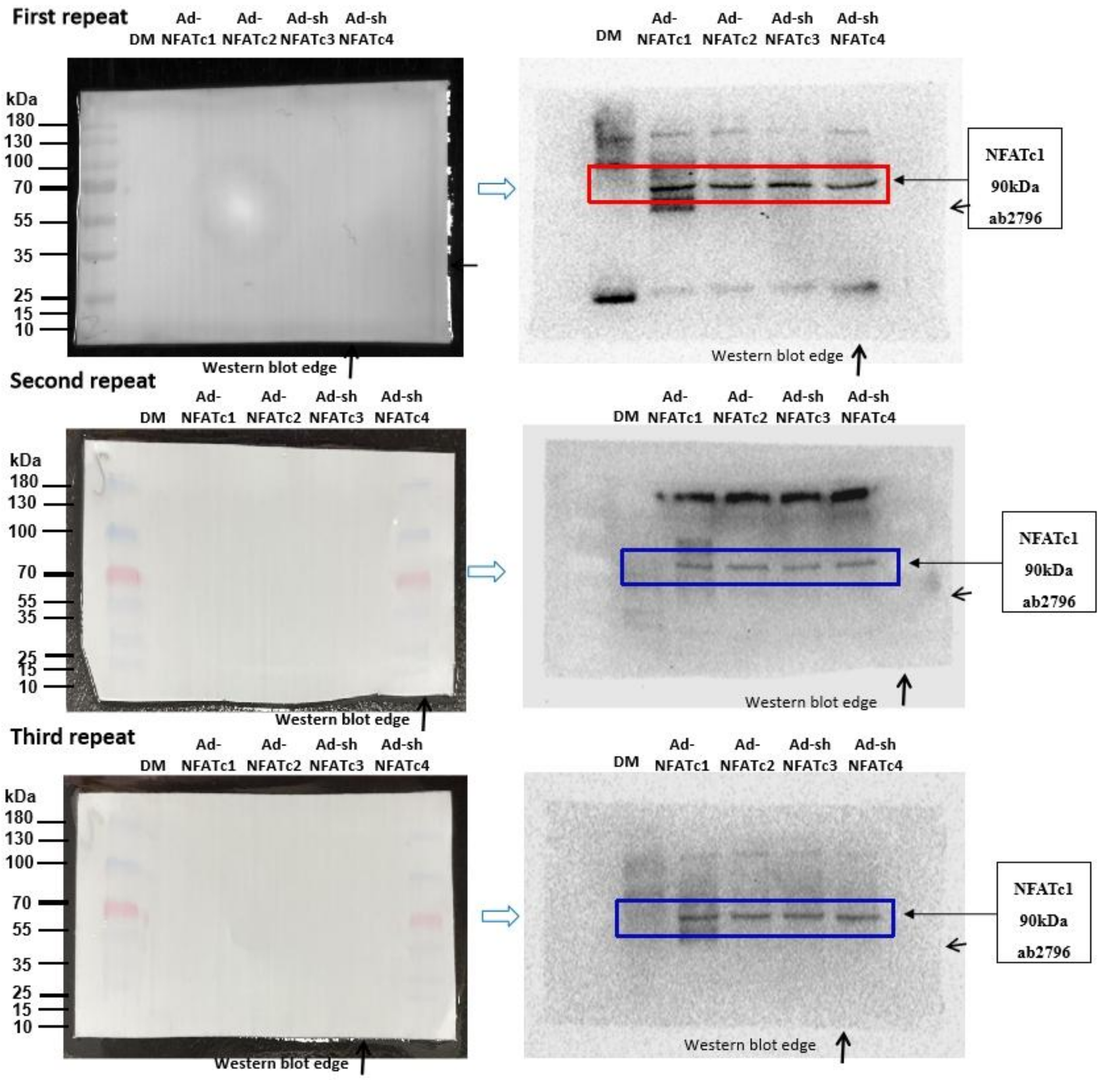

To generate this image, Lysates of myoblasts were analyzed at 6 days of differentiation. First, samples (20 µg proteins) were run on an 10 % SDS-PAGE gel then transferred onto a PVDF membrane (Millipore). Membranes were blocked in 5% nonfat milk in TBS-0.1 % Tween® 20 (TBS-T) before incubation with NFATc1-mouse anti-mouse/human (1:500, ab2796,ABCAM,USA) antibody overnight at 4 ℃, respectively. Blots were washed four times in TBS-T, incubated with secondary antibodies for 90 min at room temperature, washed again four times then imaged. Secondary antibodies used were goat anti-mouse IgG H&L(ANT019, antgene.CN) preabsorbed at 1/2000 dilution.

In order to save samples and workload, we divide the glue and film into two, as shown in the figure. Predicted band size: 90 kDa. Observed band size: 75,80,90 kDa.

https://www.abcam.cn/nfat2-antibody-7a6-ab2796.html

Figure 5H for NFATc2: the result of three replicates.

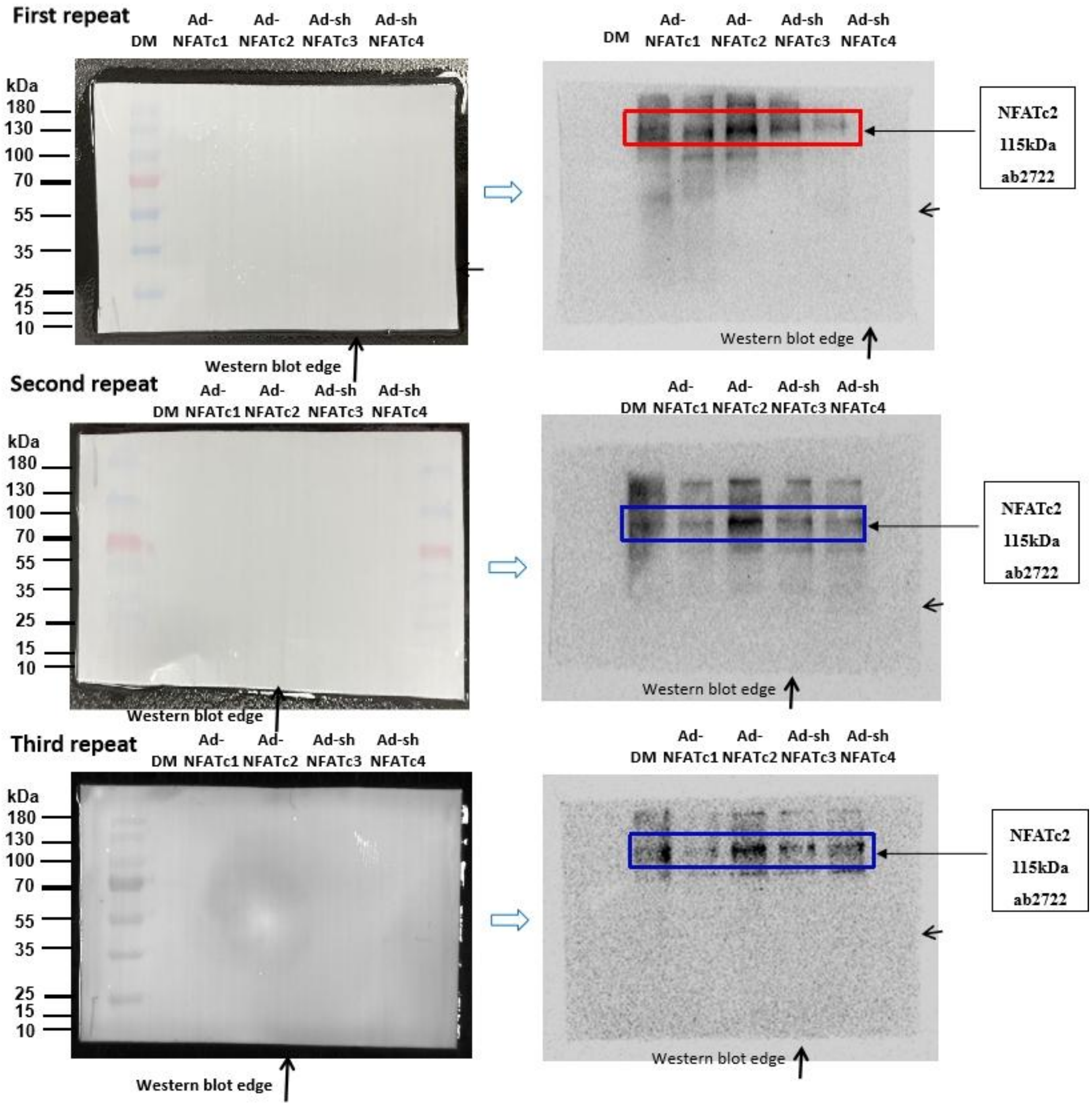

To generate this image, Lysates of myoblasts were analyzed at 6 days of differentiation. First, samples (20 μg proteins) were run on an 10 % SDS-PAGE gel then transferred onto a PVDF membrane (Millipore). Membranes were blocked in 5% nonfat milk in TBS-0.1 % Tween® 20 (TBS-T) before incubation with NFATc2-mouse anti-mouse/human (1:500, ab2722,ABCAM,USA) antibody overnight at 4 ℃, respectively. Blots were washed four times in TBS-T, incubated with secondary antibodies for 90 min at room temperature, washed again four times then imaged. Secondary antibodies used were goat anti-mouse IgG H&L(ANT019, antgene.CN) preabsorbed at 1/10000 dilution.

Predicted band size: 115 kDa; Observed band size: 150 kDa.

https://www.abcam.cn/nfat1-antibody-25a10d6d2-ab2722.html

Figure 5H for NFATc3: the result of three replicates.

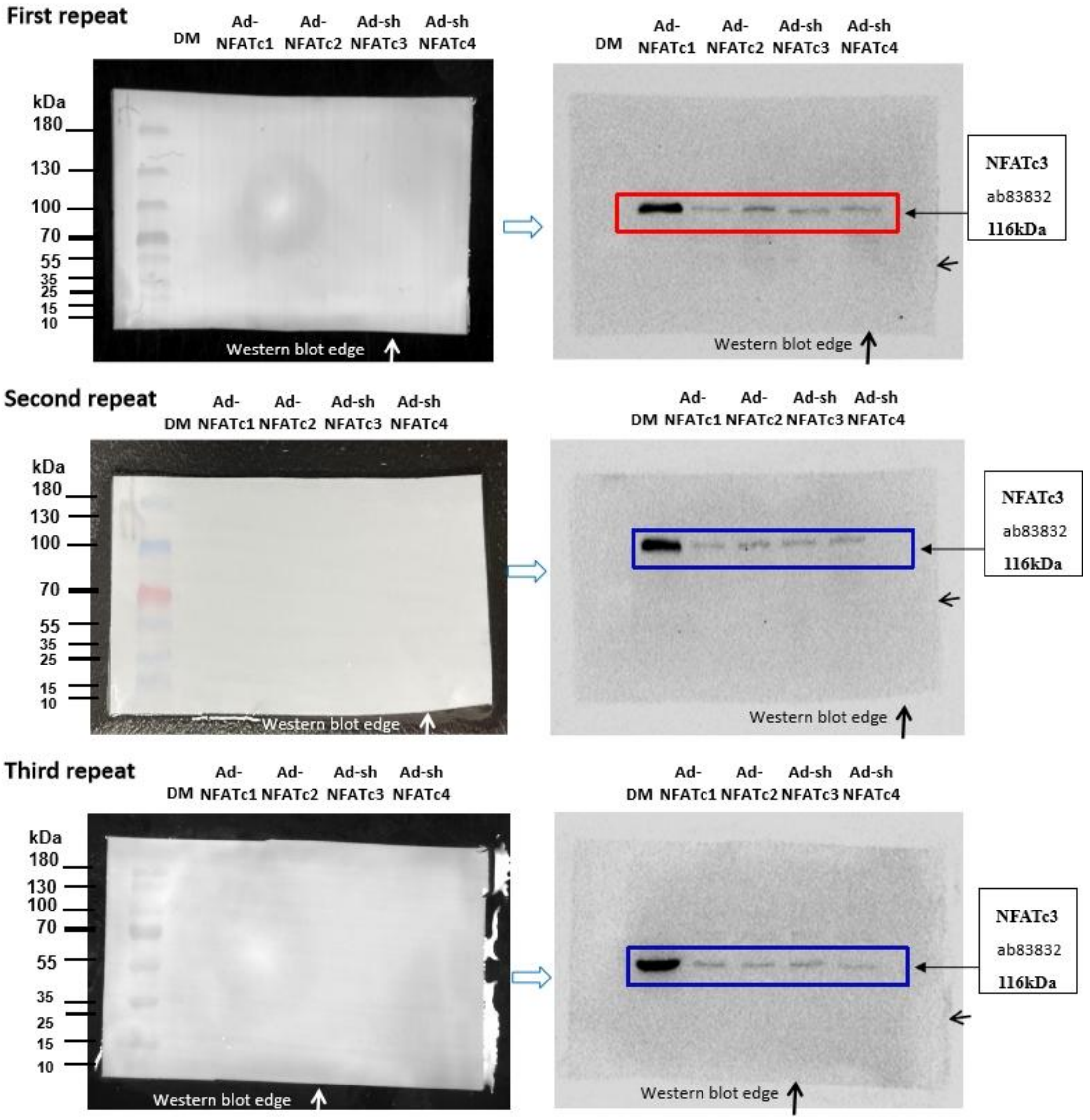

To generate this image, Lysates of myoblasts were analyzed at 6 days of differentiation. First, samples (20 μg proteins) were run on an 10 % SDS-PAGE gel then transferred onto a PVDF membrane (Millipore). Membranes were blocked in 5% nonfat milk in TBS-0.1 % Tween® 20 (TBS-T) before incubation with NFATc3 rabbit anti-mouse/human (1:500, ab83832,ABCAM,USA) antibody overnight at 4 ℃, respectively. Blots were washed four times in TBS-T, incubated with secondary antibodies for 90 min at room temperature, washed again four times then imaged. Secondary antibodies used were goat anti-rabbit IgG H&L (ANT020, antgene.CN) preabsorbed at 1/2000 dilution.

Observed band size for NFATc3: 116 kDa.

https://www.abcam.cn/nfat4nf-atc3-antibody-ab83832.html

Figure 5H for NFATc4: the result of three replicates.

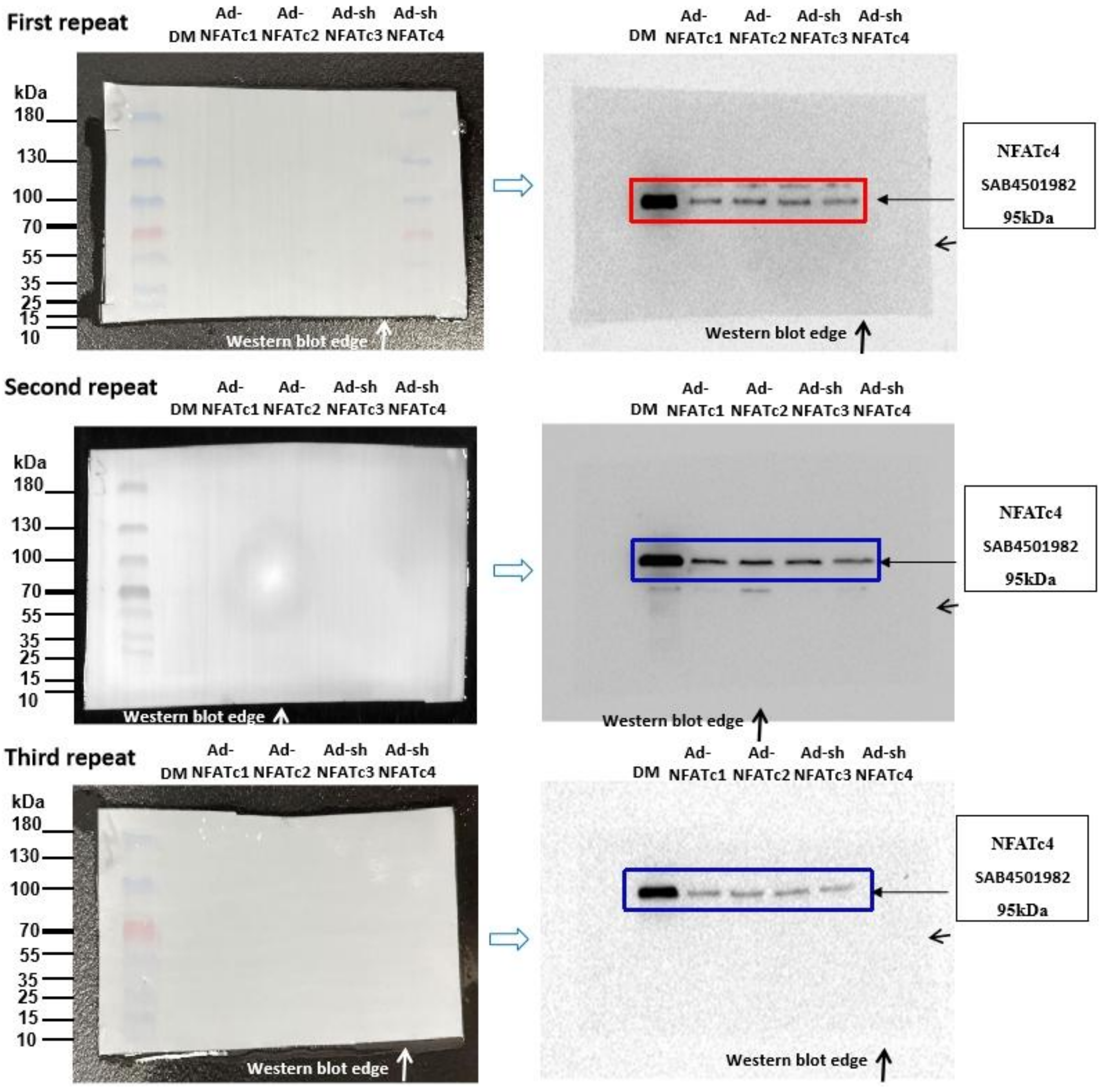

To generate this image, Lysates of myoblasts were analyzed at 6 days of differentiation. First, samples (20 μg proteins) were run on an 10 % SDS-PAGE gel then transferred onto a PVDF membrane (Millipore). Membranes were blocked in 5% nonfat milk in TBS-0.1 % Tween® 20 (TBS-T) before incubation with NFATc4 rabbit anti-mouse/human (1:1000,ab99431, ABCAM, USA; 1:1000, SAB4501982, SIGMA, USA) antibody overnight at 4 ℃, respectively. Blots were washed four times in TBS-T, incubated with secondary antibodies for 90 min at room temperature, washed again four times then imaged. Secondary antibodies used were goat anti-rabbit IgG H&L (ANT020, antgene.CN) preabsorbed at 1/10000 dilution.

Observed band size for NFATc4: 95 kDa.

https://www.sigmaaldrich.cn/CN/zh/product/sigma/sab4501982

Figure 5H for LaminB1: the result of three replicates.

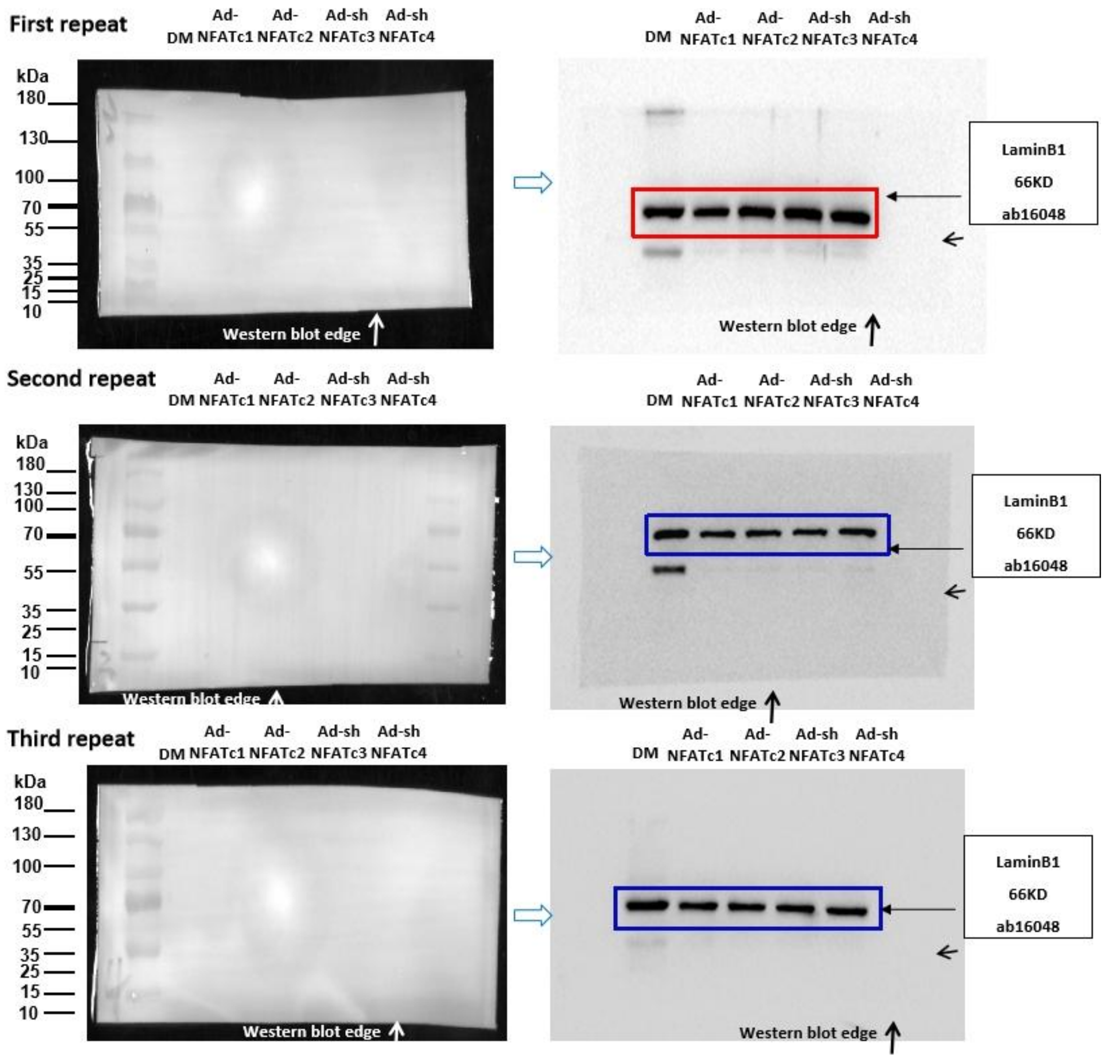

To generate this image, Lysates of myoblasts were analyzed at 6 days of differentiation. First, samples (20 μg proteins) were run on an 10 % SDS-PAGE gel then transferred onto a PVDF membrane (Millipore). Membranes were blocked in 5% nonfat milk in TBS-0.1 % Tween® 20 (TBS-T) before incubation with Lamin B1 rabbit anti-mouse/human (1:1000, ab16048, ABCAM, USA; 1:1000) antibody overnight at 4 ℃, respectively. Blots were washed four times in TBS-T, incubated with secondary antibodies for 90 min at room temperature, washed again four times then imaged. Secondary antibodies used were goat anti-rabbit IgG H&L (ANT020, antgene.CN) preabsorbed at 1/10000 dilution.

Observed band size: 73 kDa;Additional bands: 46 kDa.

https://www.abcam.cn/lamin-b1-antibody-nuclear-envelope-marker-ab16048.html
