# Supplementary material for: Continuous exposure to isoprenaline reduced myotube size by delaying myoblast differentiation and fusion through the NFAT-MEF2C signaling pathway
Source: Sci Rep. 2023 Jan 9;13:436. doi: 10.1038/s41598-022-22330-w (PMC9829891; doi:10.1038/s41598-022-22330-w)
Supplement: Supplementary file 2 — Supplementary Information 2. [file 41598_2022_22330_MOESM2_ESM.pdf]

Continuous exposure to isoprenaline reduced myotube size by delaying myoblast differentiation and fusion through the NFAT-MEF2C signaling pathway

Jing Yue[1, 2#], Wei Xu[1,3#], Li Xiang [1,3], Shao-juan Chen[4], Xin-yuan Li[5], Qian Yang[1,3], Ruo-nan Zhang[1,3], Xin Bao[1,3], Yan Wang[1,3], MagdaleenaNaemi Mbadhi[1,3], Yun Liu[1,3], Lu-yuan Yao[1,3], Long Chen[6], Xiao-ying Zhao[1,3], Chang-qingHu[1,3],Jing-xuan Zhang[1,3], Hong-tao Zheng[1,3], Yan Wu[1,3], Shi-You Chen[7], Shan Li[8], Jing Lv[9*], Liu-liu Shi[1,3*], Jun-mingTang[1, 3*]

[1]Department of Physiology, Faculty of Basic Medical Sciences, Hubei University of Medicine, Shiyan, Hubei 442000, PR China

[2]Continuing Education Department, Affiliated Hospital of Guilin Medical University, Guilin, Guangxi 541000, PR China

[3]Hubei Key Laboratory of Embryonic Stem Cell Research and Institute of Biomedicine, Hubei University of Medicine, Hubei, 442000, China.

[4]Department of Stomatology, Taihe Hospital, Hubei University of Medicine, Shiyan, Hubei 442000, PR China

[5]Department of Physiology, Faculty of Basic Medical Sciences, Zunyi Medical University, Zunyi, Guizhou563006, PR China

[6]Experimental Medical Center, Dongfeng Hospital, Hubei University of Medicine, Shiyan, China.

7Department of Surgery, University of Missouri, Columbia, U.S.A.

[8]Department of Biochemistry, Faculty of Basic Medical Sciences, Hubei University of Medicine, Shiyan, 442000, Hubei, People's Republic of China.

[9]Department of Anesthesiology, Taihe Hospital, Hubei University of Medicine, Shiyan, Hubei 442000, PR China

[#]Co-first author

Corresponding Author: Jing Lv, Liu-liu Shi & Jun-ming Tang

Department of Physiology, Faculty of Basic Medical Sciences, Hubei University of Medicine, Shiyan, Hubei 442000, PR China

Phone: 86-0719-8875312, Email: 389514970@qq.com(Lv), shi-liuliu@163.com(Shi) &tangjm416@163.com (Tang)

Supplemental.Figure.1. NFAT involved in the inhibition of C2C12 myoblast cell differentiation and myoblast fusion by ISO

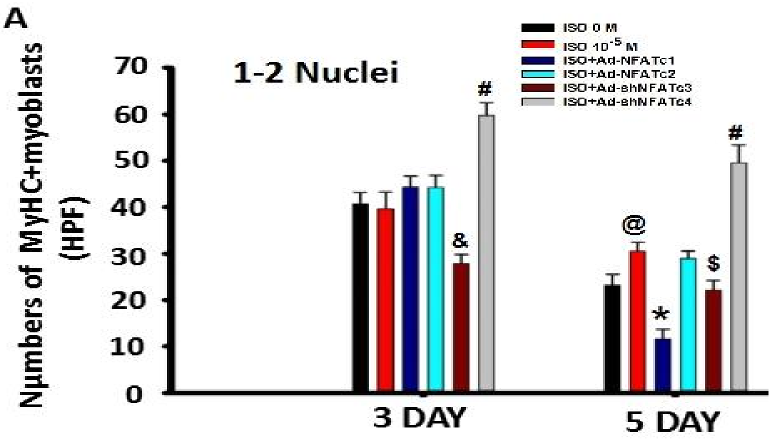

sFigure1. NFAT involved in the inhibition of C2C12 myoblast cell differentiation and myoblast fusion by ISO. (A)Myoblast cells were transfected with indicated adenovirus for 24h, prior to stimulation of continuous single-dose ISO for three and five days. Then quantitative assay for the number of MyHC+ cells with 1-2 nuclei from results figure.6. n=3, $^{@}P < 0.05$ vs. Ctrl;$^{\&}P < 0.05$ vs. 0M ISO, 10^{-5} M ISO, ISO+Ad-NFATc1, ISO+Ad-NFATc12 or ISO+AdshNFATc4 group; $^{\#}P < 0.05$ vs. all groups; $^{*}P < 0.05$ vs. all groups.

Supplemental.Figure2.NFATc4 overexpression involved in the partial recovery of myoblast differentiation and myoblast fusion by ISO

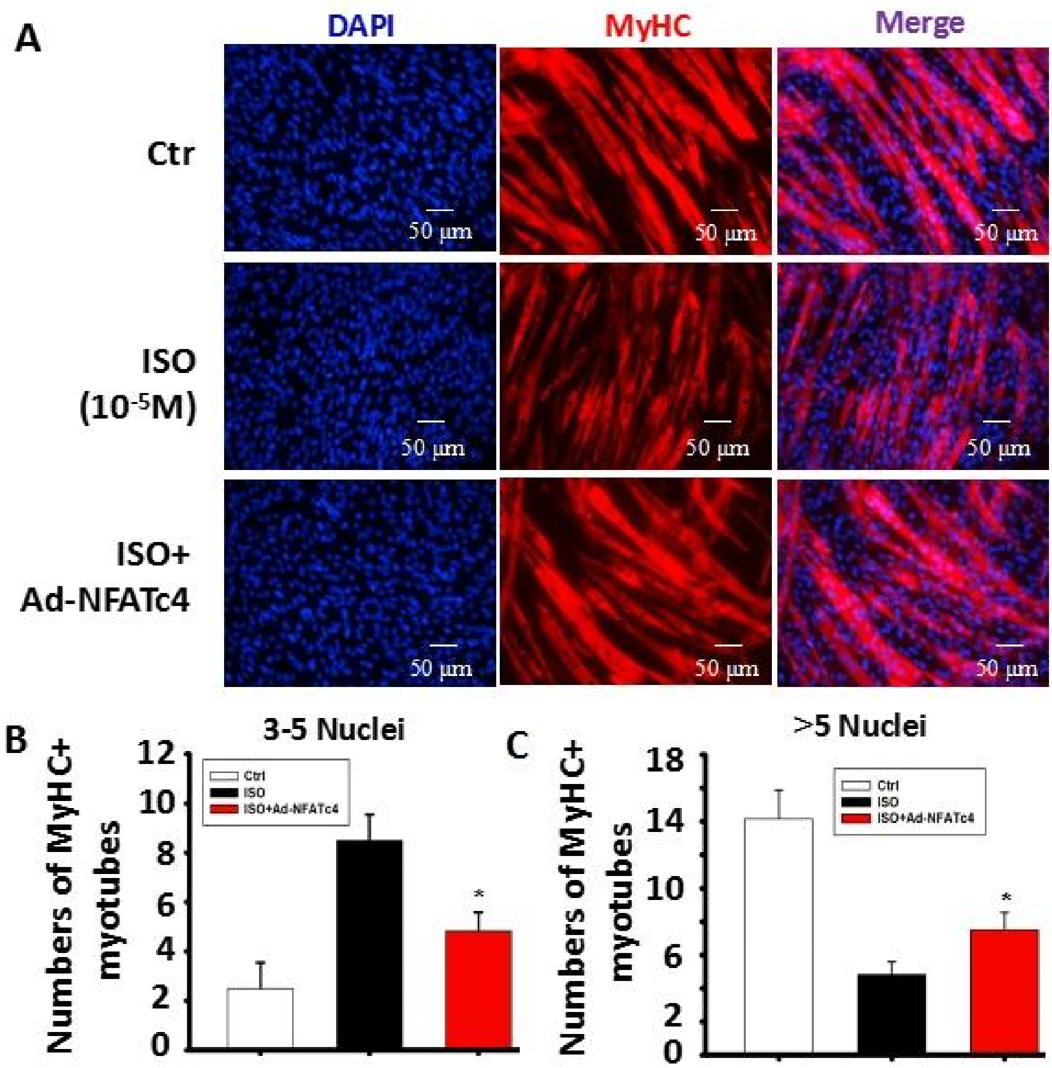

sFigure2. NFATc4 overexpression involved in the partial recovery of myoblast differentiation and myoblast fusion by ISO. (A) Myoblast cells were stimulated by continuous single-dose ISO under differentiation medium for three and five days, and respectively stained by MyHC, 24h afte rthe cells were transfected with adenovirus mediated over-expression of NFATc4 (Ad-NFATc4) with 100 MOI. Three independently repeated experiments were done. (B) Quantitative assay for the number of MyHC+ myotubes with 3-5 or more than 5 nuclei from sFigure 2A. n=3,$^{*}P<0.05$ vs. ISO group.
